# DDX21 Promotes Breast Cancer Growth and Metastasis via Stimulating RNAPII Elongation During Hypoxia

**DOI:** 10.1002/mco2.70792

**Published:** 2026-06-15

**Authors:** Guangqiang Li, Mingxia Deng, Leqing Zhu, Zhiwei Lei, Rong Guo, Xiong Liu, Renwang Chen, Xichun Xia, Qiong Wen, Yuanyuan Duan, Yan Chen, Zhinan Yin

**Affiliations:** ^1^ The Biomedical Translational Research Institute, Health Science Center (School of Medicine) Jinan University Guangzhou China; ^2^ Guangzhou National Laboratory Guangzhou International BioIsland Guangzhou China; ^3^ Department of Gastroenterology, Affiliated Qingyuan Hospital Guangzhou Medical University, Qingyuan People's Hospital Qingyuan China; ^4^ The First Affiliated Hospital of Gannan Medical University Ganzhou China; ^5^ Clinical Laboratory Hunan Aerospace Hospital Changsha China; ^6^ Institute of Dermatology and Venereal Diseases, Dermatology Hospital Southern Medical University Guangzhou China; ^7^ Key Laboratory of Viral Pathogenesis & Infection Prevention and Control (Jinan University), Ministry of Education, School of Medicine Jinan University Guangzhou China; ^8^ Center For Cell Structure and Function, Institute of Biomedical Science, College of Life Sciences Shandong Normal University Jinan China; ^9^ The Affiliated Guangdong Second Provincial General Hospital of Jinan University Guangzhou China

**Keywords:** breast cancer, DDX21, hypoxia, RNA helicase, transcription elongation

## Abstract

Hypoxia is a hallmark of solid tumors, and hypoxia‐inducible factors (HIFs) are the master transcription factors that allow cancer cells to sense and cope with a hypoxic microenvironment. However, the mechanism by which the HIF‐mediated transcription process is precisely regulated remains poorly understood. Here, we demonstrate that the RNA helicase DDX21 facilitates breast tumor growth and metastasis by triggering HIF‐mediated transcription elongation. Mechanistically, DDX21 induces the release of CDK9 from the 7SK complex, enhancing the interaction between CDK9 and HIF‐1α. This interaction stimulates the transcription elongation of HIF target genes by elevating the enrichment of phosphorylated RNA Polymerase II at serine 2 on the hypoxia‐response element of HIF target genes. These target genes, including IGFBP1, WNT1, WNT10B, TGFB1, PCK1, and SLC2A3, are critical for cancer cell proliferation and metastasis. Consequently, DDX21 enhances breast cancer cell proliferation, survival, migration, and invasion in vitro, and promotes breast tumor growth and metastasis in vivo. Importantly, DDX21 is highly expressed in breast tumors and correlates negatively with clinical outcomes in breast cancer patients. Our study reveals a novel mechanism of HIF regulation and positions DDX21 as a potential therapeutic target for disrupting the hypoxic adaptation machinery in breast cancer.

## Graphical abstract

DDX21 triggers CDK9 release from the 7SK RNP complex.

DDX21‐CDK9 binding to HIFs drives transcriptional reprogramming during hypoxia.

DDX21 enhances breast cancer progression and metastasis.

Elevated DDX21 expression correlates with poor prognosis in breast cancer patients.

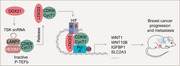

## 1 Introduction

Breast cancer (BRCA) is the most prevalent malignant tumor among women, with metastasis accounting for over 90% of BRCA‐related deaths [1]. Intratumoral hypoxia is a hallmark feature of the vast majority of solid tumors, with rapid tumor metabolism and tissue compression leading to a hypoxic microenvironment, activating hypoxia‐inducible factors (HIFs) to promote tumor growth, invasion and metastasis, and patient mortality [2, 3]. The heterodimeric transcription factor composed of an oxygen‐sensitive HIF‐α subunit (HIF‐1α, HIF‐2α, or HIF‐3α) and a constitutively expressed HIF‐1β subunit mediates the expression of hundreds of genes to adapt to oxygen stress [4]. Under normoxic conditions, prolyl hydroxylases catalyze O_2_‐dependent hydroxylation of proline residues in HIF‐α, which is then recognized by the tumor suppressor VHL and targeted for ubiquitination and degradation [5]. In contrast, hypoxia stabilizes HIF‐α proteins by inhibiting the hydroxylation of HIF‐α. Accumulated HIF‐α forms a dimer with HIF‐1β, translocates into the nucleus, binds to the hypoxia‐response element (HRE) of target genes, and recruits the transcription coactivator p300 to further stimulate the transcription of target genes [6, 7]. HIF target genes are involved in various biological processes, including angiogenesis, tumor metastasis, metabolic regulation, drug resistance, and others [8].

The stability and transcriptional activity of HIFs are tightly regulated by a multilevel control network. While the classical oxygen‑dependent pVHL‑mediated degradation pathway is well characterized [9], noncanonical regulatory pathways are more complex and less defined. Posttranslational modifications of HIF‑1/2α are known to be crucial: for instance, acetylation/deacetylation by ARD1 [10], MAT1 [11], and SIRT1 [12, 13], dynamically modulate HIF stability and activity; SUMOylation also participates in regulating HIF stability. Simultaneously, epigenetic regulators are also involved in modulating HIF function: G9a [14] represses HIF transcriptional activity through methylation, whereas LSD1 [15] inhibits HIF degradation via demethylation, thereby promoting HIF stability. Furthermore, upon binding to target gene DNA, HIF acts as a regulatory hub, recruiting coregulatory factors such as p300/CBP [16, 17], TIP60 [18], chromatin remodelers (e.g., JMJD2C [19], JMJD1A [20]), and epigenetic modifiers (e.g., SETDB1[21], PADI4 [22]) to cooperatively drive target gene transcription. The specific mechanisms by which these factors participate in transcriptional machinery recruitment and activity regulation still require further investigation.

DEAD‐box RNA helicases are vital for the regulation of various aspects of the RNA life cycle [23]. DDX21, also known as RNA helicase II/Gu, initially purified from HeLa nuclei, exhibits ATP/poly(C)‐dependent 5′–3′ unwinding activity and induces ssRNA hairpin formation to modulate RNA dynamics [24]. DDX21 plays pivotal roles in diverse developmental processes and disease regulation [25], including modulating lymphangiogenesis [26], orchestrating innate immune responses [27], and maintaining genomic homeostasis [28]. DDX21 dysregulation is frequently observed in human cancers and exhibits multiple roles in tumorigenesis. Studies demonstrate that DDX21 functions as an oncogene in gastric cancer [29, 30], colorectal cancer [31], neuroblastoma [32], and BRCA [33]. However, other research has identified DDX21 as a metastasis suppressor in BRCA [34]. Furthermore, as a multifunctional RNA‐binding protein, DDX21 senses the transcriptional status of both RNA Polymerase (Pol) I and II to regulate multiple steps of gene expression through genomic stability, transcription, posttranscriptional modifications, and RNA metabolism [25].

Research has shown that SIRT7 colocalizes with DDX21 at RNA Pol I and II (Pol II) transcription machinery, where the deacetylase SIRT7 dynamically regulates acetylation status of DDX21 to maintain genomic stability [35]. In the nucleolus, DDX21 occupies the transcribed rDNA locus and facilitates the transcription, processing, and modification of rRNA. In the nucleoplasm, DDX21 binds to 7SK RNA and is recruited to the promoters of RNA Pol II‐transcribed genes to facilitate the release of the positive transcription elongation factor b (P‐TEFb) from the 7SK snRNP complex, thereby enhancing the transcription of its target genes, which encode ribosomal proteins and snoRNAs [36]. Additionally, DDX21 binds to noncoding RNAs to regulate transcription and posttranscriptional modifications, thereby promoting tumorigenesis [37]. However, the role of DDX21 in RNA Pol II‐mediated mRNA transcription regulation remains elusive. Notably, P‐TEFb plays a crucial role in promoting transcription elongation, especially for inducible gene expression. The activity of P‐TEFb is tightly regulated, with three main complexes identified in mammals: the 7SK small nuclear ribonucleoprotein (7SK RNP), the bromodomain protein 4 (BRD4), and the super elongation complex (SEC) or SEC‐like complexes [38]. While studies have revealed both SEC [39] and BRD4 complexes [40] regulate P‐TEFb activity in hypoxia to promote the release of paused RNA Pol II from HIF target gene promoters, whether the 7SK complex controls HIF‐mediated transcriptional elongation is still unknown.

To address these gaps, this study aimed to investigate whether DDX21 influences HIF‐mediated transcriptional elongation by modulating the 7SK complex, thereby contributing to BRCA progression. We found DDX21 induces the release of CDK9 from the 7SK complex, enhancing its interaction with HIF‐1α, increasing the enrichment of phosphorylated RNA Pol II (Pol II‐S2P) on HIF target genes, and promoting the transcriptional elongation of HIF target genes, ultimately promoting breast tumor growth and metastasis. This study advances our understanding of hypoxic tumor microenvironment regulation, suggests DDX21 as a potential BRCA therapeutic target, and offers new research avenues and scientific support for targeting HIF‐driven cancer progression.

## 2 Results

### 2.1 DDX21 Interacts with HIFs

**FIGURE 1:**
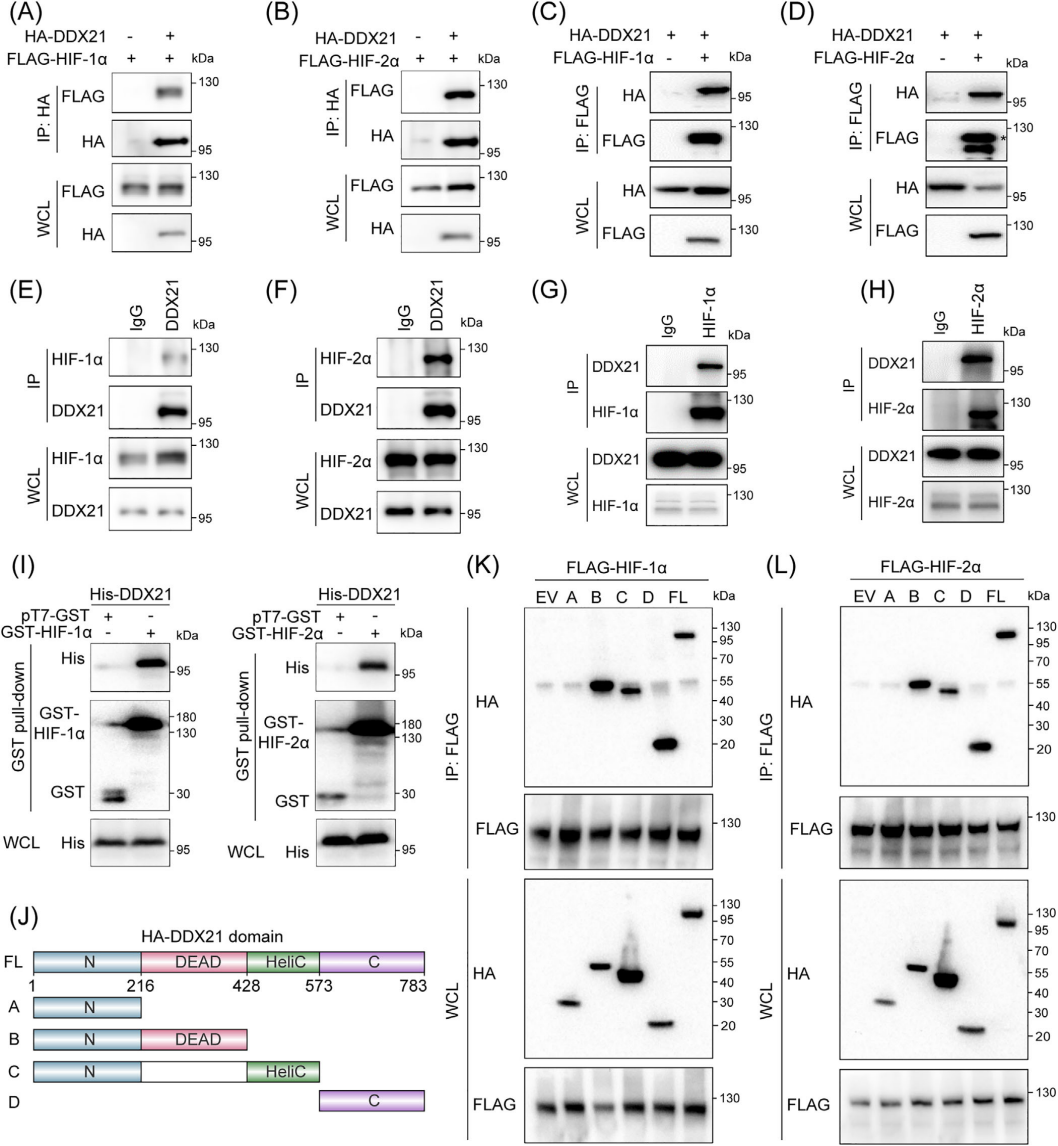
DDX21 interacts with HIF‐1α and HIF‐2α. (A–D) HA–DDX21 and FLAG–HIF‐1α (A and C) or HA–DDX21 and FLAG–HIF‐2α (B and D) were cotransfected in HEK293T cells and then subjected to co‐IP assays of exogenous DDX21 and HIF‐1α or HIF‐2α (* indicates the target FLAG band). (E–H) Co‐IP assays of endogenous DDX21 and HIF‐1α or HIF‐2α with anti‐DDX21 (E and F), anti‐HIF‐1α (G), or anti‐HIF‐2α (H) antibody in MDA‐MB231 cells exposed to 1% O_2_ for 6 h. (I) GST pull‐down and IB analyses were performed using the purified GST‐HIF‐1α, GST‐HIF‐2, and His‐DDX21α in vitro. (J–L) Schematic diagram of DDX21 domain (J). Domain mapping of DDX21 binding to HIF‐1α (K) and HIF‐2α (L). *Abbreviation*: WCL, whole cell lysate.

Hypoxia is a key regulatory factor in tumor growth [41] and metastasis [2] and a driving force for BRCA progression [21]. HIFs are the master transcription factors that enable cancer cells to sense and adapt to hypoxic microenvironment [42]. However, the mechanism by which the HIF‐mediated transcription process is precisely regulated remains poorly understood. Recent studies have revealed that several RNA helicases play crucial roles in hypoxia adaptation through crosstalk with HIFs [43]. To screen for RNA helicases that interact with HIF, we cotransfected HA‐tagged RNA helicases (DDX19A, DDX21, DHX29, DHX32, DDX41, DDX49, EIF4A1, EIF4A2, EIF4A3) individually​ with FLAG‐tagged HIF‐1α into HEK293T cells and assessed interactions by co‐immunoprecipitation (co‐IP). The results confirmed that DDX21, DHX32, DDX41, and EIF4A3 all physically interact with HIF‐1α (Figure S1A–D) with notably stronger binding observed for DDX21 and DDX41. Given the multifaceted roles of DDX21 in various biological processes, including RNAPII‐mediated transcriptional regulation [36], we focused on DDX21 to investigate its function in HIF‐mediated transcriptional processes. To determine whether DDX21 interacts with both HIF‐1α and HIF‐2α under hypoxic conditions, we performed co‐IP assays in HEK293T cells coexpressing HA‐tagged DDX21 and FLAG‐tagged HIF‐1α or HIF‐2α under hypoxic conditions (1% O_2_, 6 h). The results showed that HA–DDX21 could effectively pull down FLAG–HIF‐1α (Figure 1A) and FLAG–HIF‐2α (Figure 1B). We also conducted reciprocal co‐IP assays using anti‐FLAG antibody, which confirmed the interaction between HA–DDX21 and both FLAG–HIF‐1α and FLAG–HIF‐2α in HEK293T cells (Figure 1C,D).

To substantiate the physical interaction between DDX21 and HIF‐1α or HIF‐2α in human BRCA cells, endogenous co‐IP assays were conducted using an anti‐DDX21 antibody, which demonstrated that DDX21 could pull down endogenous HIF‐1α (Figure 1E) and HIF‐2α (Figure 1F). In addition, reciprocal endogenous co‐IP assay using anti‐HIF‐1α (Figure 1G) or anti‐HIF‐2α (Figure 1H) antibody confirmed that HIF‐1α or HIF‐2α pulled down DDX21 in hypoxic MDA‐MB231 cells. Similar results were observed in MCF‐7 cells (Figure S2A–D). In addition, GST pull‐down assays indicated that DDX21 can directly interact with both HIF‐1α and HIF‐2α (Figure 1I). Collectively, these findings confirm that DDX21 physically interacted with both HIF‐1α and HIF‐2α under hypoxia.

To further elucidate the binding domains of DDX21 and HIFs, we constructed vectors encoding each domain of DDX21 (Figures 1J and S2E), and then mapped the DDX21 domain binding to HIF‐α. Vectors encoding each HA‐tagged domain of DDX21 and FLAG–HIF‐1α or FLAG–HIF‐2α were cotransfected in HEK293T cells and were exposed to 1% O_2_ for 6 h, followed by co‐IP. The results revealed that three distinct domains of DDX21, the DEAD domain, Helicase domain (HeliC), and C‐terminal domain, independently bound HIF‐1α (Figures 1K and S2F) and HIF‐2α (Figures 1L and S2G).

### 2.2 DDX21 Regulates the Expression of Specific HIF Target Genes

**FIGURE 2:**
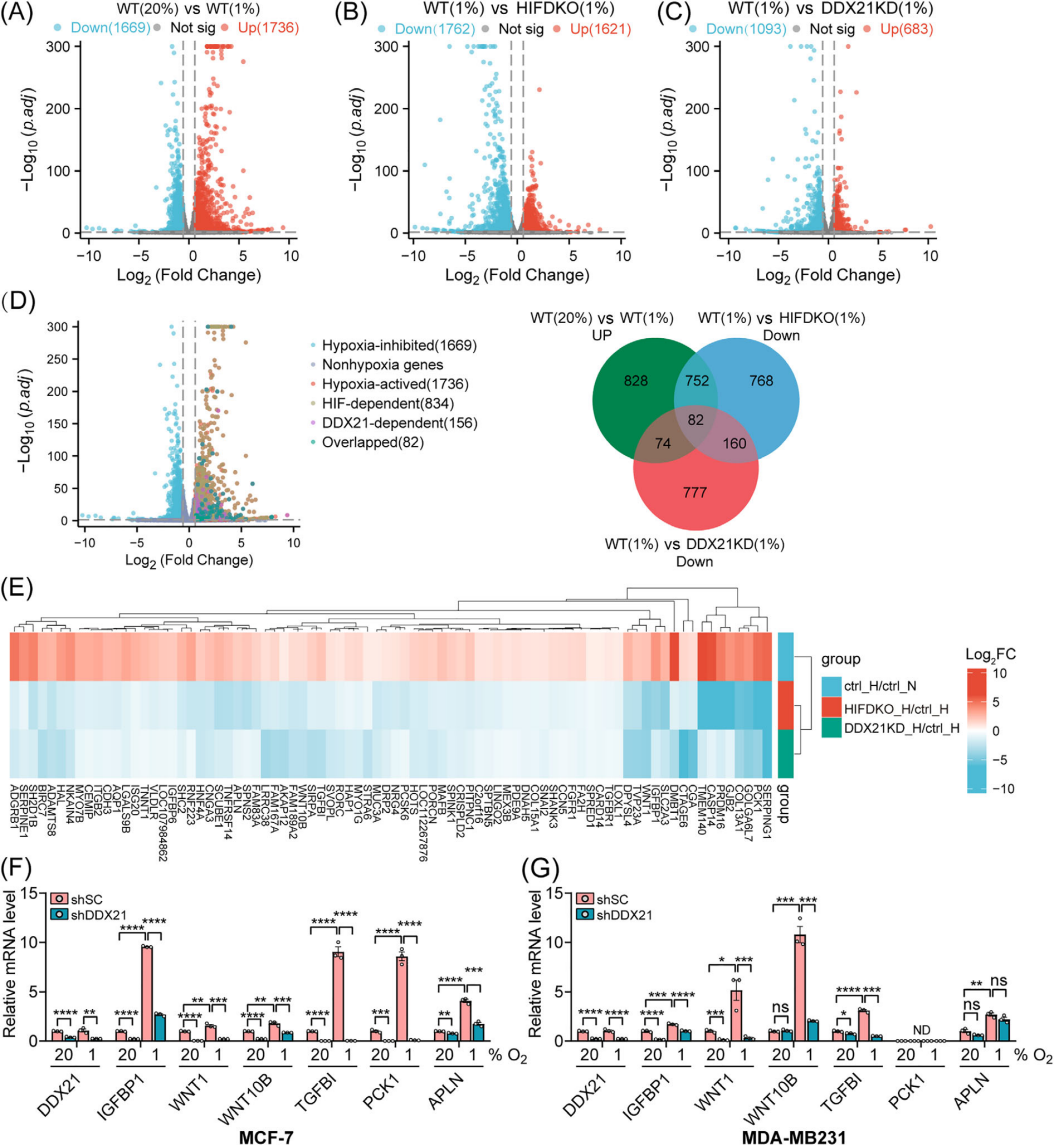
DDX21 promotes HIFs‐specific target gene expression. (A–C) Volcano plots were generated to visualize global transcriptional changes between WT (20%) versus WT (1%) (A), WT (1%) versus HIFDKO (1%) (B), WT (1%) versus DDX21KD (1%) (C). (D) Multigroup volcano plots of hypoxia‐responsive genes stratified by HIF and DDX21 dependency. (E) Heatmap displaying differentially expressed genes after DDX21 knockdown and HIF knockout. (F and G) RNA‐sequencing results were validated by qPCR in MCF‐7 (F) and MDA‐MB231 (G) cell lines (*n* = 3, mean ± SEM, *p < 0.05, **p < 0.01, ***p < 0.001, ****p < 0.0001, ns, not significant).

To systematically investigate the role of DDX21, we performed transcriptome sequencing on DDX21‐knockdown MCF‐7 cells under both hypoxic and normoxic conditions. RNA‐sequencing (RNA‐seq) analysis revealed significant transcriptional remodeling: 1669 genes were downregulated (*p* < 0.05, |FC| ≥ 1.5) while 1736 genes were markedly upregulated (Figure 2A). HIFs knockout led to comparable transcriptional alterations, downregulating 1762 genes and upregulating 1621 genes (Figure 2B). Notably, DDX21 knockdown under hypoxia resulted in distinct gene expression profiles, with 1093 genes downregulated and 683 genes upregulated (Figure 2C). Integrated RNA‐seq analysis revealed 1736 hypoxia‐activated genes, of which 834 were HIF‐dependent and 156 were DDX21 dependent. Moreover, 82 genes exhibited dual dependency on both HIFs and DDX21 (Figure 2D). These overlapping genes were functionally characterized as HIFs target genes coregulated by DDX21 (Figure 2E). We further verified the effect of DDX21 on the expression of HIF target genes in BRCA cells through quantitative polymerase chain reaction (qPCR). The results were consistent with the sequencing data (Figure 2F,G). Collectively, these findings indicate that DDX21 promoted HIF‐specific rather than global target gene expression.

### 2.3 DDX21 Promotes CDK9 Release from the 7SK Complex to Activate HIF Target Genes Transcription

To further validate the regulatory effect of DDX21 on HIF transcriptional activity, we performed dual‑luciferase reporter assays. The results showed that overexpression of DDX21 promoted HIF transcriptional activity (Figure S3A,B), while knockdown of DDX21 markedly suppressed HIF transcriptional activity (Figure S3C,D). Furthermore, in HIF‐1α/HIF‐2α double‐knockout HeLa cells, DDX21 overexpression failed to restore the hypoxia‐induced transcription of HIF target genes IGFBP1 and WNT1 (Figure S3E,F). To explore the mechanism by which DDX21 regulates HIF target gene expression, we first examined HIF‑1α and HIF‑2α protein levels in DDX21‑knockdown cells under hypoxia (24 h). DDX21 depletion did not affect HIF‑1α or HIF‑2α protein stability in MCF‐7 and MDA‐MB231 cells (Figure S4A). Cytoplasmic and nuclear fractionation assays further revealed that DDX21 knockdown did not alter HIF nuclear localization (Figure S4B). These data suggest that DDX21 regulates HIF target gene expression at the transcriptional level rather than by modulating HIF stability or nuclear translocation.

**FIGURE 3:**
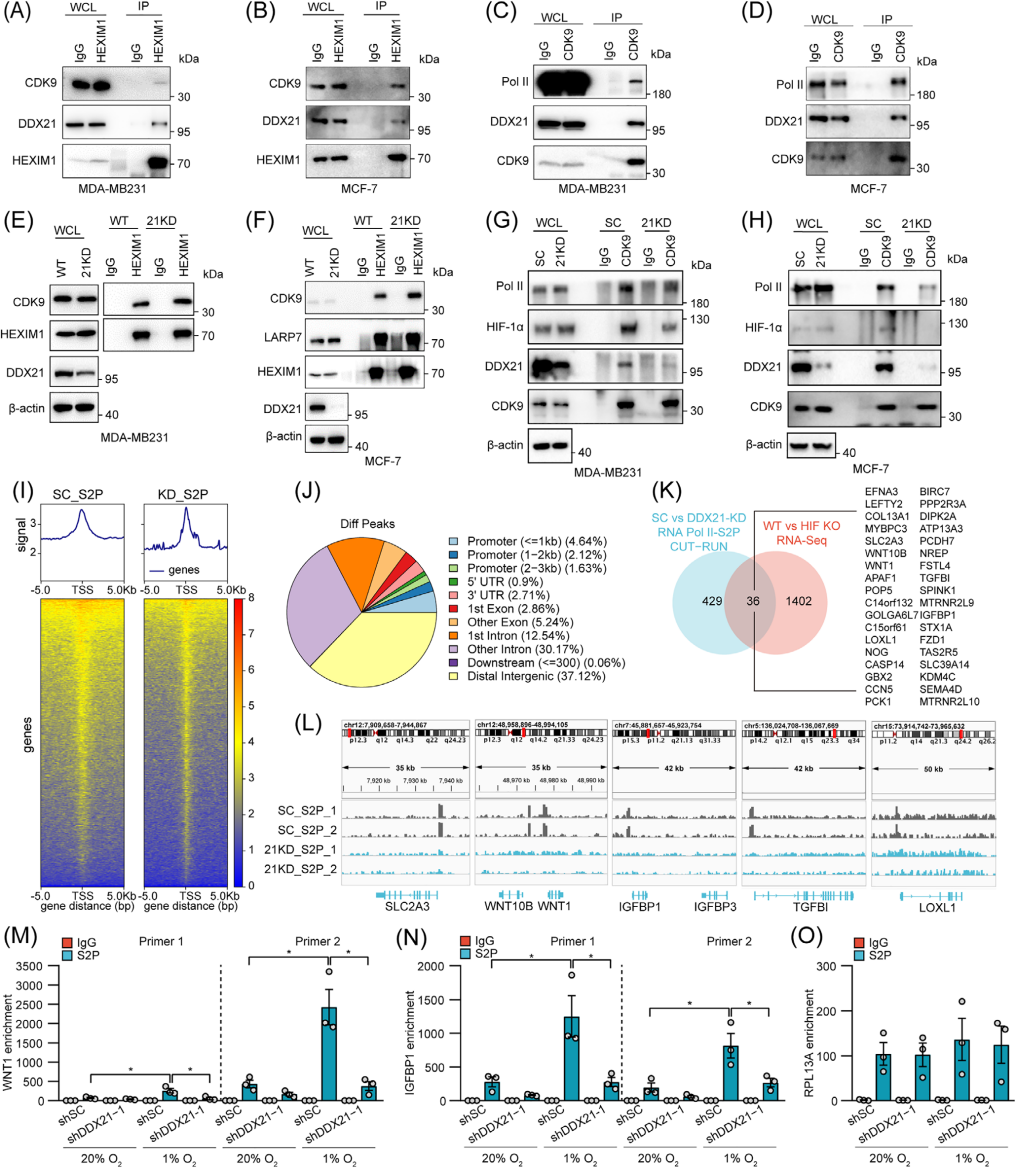
DDX21 promotes the dissociation of CDK9 from the 7SK complex. (A and B) Co‐IP analysis of the interaction between DDX21 and 7SK complex core members HEXIM1 and CDK9 in MDA‐MB231 and MCF‐7 cells. (C and D) Co‐IP analysis of the interaction between CDK9 and DDX21 as well as RNA polymerase II (Pol II) in MDA‐MB231 and MCF‐7 cells. (E and F) Co‐IP experiments were conducted in control and DDX21‐knockdown cells to study the interaction between CDK9 and HEXIM1 in MDA‐MB231 and MCF‐7. (G and H) Co‐IP experiments were performed in control and DDX21‐knockdown cells to study the interaction between CDK9 and HIF‐1α and RNA Pol II in MDA‐MB231 and MCF‐7. (I) Metagene analysis of RNA Pol II S2P genomic distribution in MDA‐MB231 and DDX21‐KD MDA‐MB231 cells treated with 1% O_2_ for 24 h (n = 2). TSS: transcription start site. (J) Pie chart showing the genomic distribution of differential peaks, with different colors indicating various genomic regions. (K) Venn diagram comparing CUT&RUN‐identified genes in SC versus DDX21‐KD samples and RNA‐seq‐identified differentially expressed genes in WT versus HIF KO samples. (L) Genome browser views of representative S2P CUT&RUN‐seq peaks. (M–O) RNA polymerase II ChIP‐qPCR analysis was performed on MDA‐MB231 cells transfected with shNC and shDDX21, which were exposed to 20 or 1% O_2_ for 24 h (*n* = 3, mean ± SEM, *p < 0.05).

Given that DDX21 binds to 7SK RNA, recruits to gene promoters, and facilitates the release of the transcription elongation factor P‑TEFb (whose core kinase is CDK9)—thereby enhancing RNA Pol II phosphorylation and promoting target gene transcription [36], we hypothesized that DDX21 might regulate CDK9 release from the 7SK complex to facilitate transcriptional elongation of HIF target genes. First, we confirmed that DDX21 could interact with CDK9 and HEXIM1 under hypoxic conditions through co‐IP (Figure 3A,B), as well as with CDK9 and RNA Pol II (Figure 3C,D). To assess whether DDX21 affects CDK9 dissociation from the 7SK complex, we performed co‑IP experiments in control and DDX21‑knockdown cells to examine CDK9–HEXIM1 binding. In three BRCA cell lines, protein levels of DDX21, CDK9, Pol II, LARP7, and HEXIM1 were not altered by oxygen concentration (Figure S4C). DDX21 knockdown did not change the expression of CDK9, Pol II, LARP7, or HEXIM1, but it increased CDK9 binding to HEXIM1 (Figure 3E,F), indicating impaired release of CDK9 from the 7SK complex upon DDX21 loss. Furthermore, DDX21 depletion reduced the binding of CDK9 to both HIF‐1α and RNA Pol II in MDA‐MB231 (Figure 3G) and MCF‐7 cells (Figure 3H). Conversely, DDX21 overexpression promoted CDK9 binding to HIF‑1α and Pol II in HEK293T cells (Figure S4D).

To determine whether DDX21 facilitates HIF‐mediated transcriptional activation by regulating the release of paused RNA Pol II, we performed RNA Pol II S2P CUT&RUN‐seq in control and DDX21‐knockdown MDA‐MB231 cells cultured under hypoxic (1% O_2_) conditions for 24 h. The results showed that S2P was highly enriched near the transcription start sites (TSS) (Figure 3I). Further analysis revealed that 8.39% of the differentially enriched peaks were located within promoter regions (−3 kb from the TSS) (Figure 3J). Among the 465 genes with significant S2P enrichment, 36 overlapped with DDX21‐dependent HIF target genes (Figure 3K). Representative genome browser views of S2P CUT&RUN‐seq peaks are shown in Figure 3L. Notably, S2P enrichment at these HIF target genes—including SLC2A3, WNT10B, WNT1, IGFBP1, TGFBI, and LOXL1—was markedly reduced upon DDX21 knockdown. To validate these findings, we further performed S2P ChIP‐qPCR in control and DDX21‐knockdown MDA‐MB231 cells under both normoxic (20% O_2_) and hypoxic (1% O_2_) for 24 h. The results demonstrated that hypoxia significantly increased RNA Pol II‐S2P occupancy at the WNT1 and IGFBP1 loci (Figure 3M,N). Importantly, DDX21 knockdown markedly decreased RNA Pol II‐S2P enrichment at these HIF target genes under hypoxia, while having no effect on the housekeeping gene RPL13A (Figure 3O). Collectively, these findings demonstrate that DDX21 facilitates Pol II‐S2P recruitment to HIF target genes, thereby promoting HIF‐mediated transcriptional activation.

### 2.4 DDX21 Increases Hypoxia‐Driven Growth and Invasion of BRCA Cells

To investigate the role of DDX21 in hypoxia‐driven tumor progression, we analyzed its expression correlation with hypoxia signatures in BRCA samples from the TCGA database. DDX21 expression showed a strong positive correlation with hypoxia scores derived from the HARRIS_HYPOXIA gene set (Figure S5A), BUFFA_HYPOXIA_METAGENE gene set (Figure S5B), and WINTER_HYPOXIA gene set (Figure S5C). Consistent with this, single‑cell transcriptomic data from CancerSEA revealed significant associations between DDX21 expression and hypoxia‐related biological processes (Figure S5D), as well as prometastatic phenotypes such as invasion (Figure S5E) and metastasis (Figure S5F).

**FIGURE 4:**
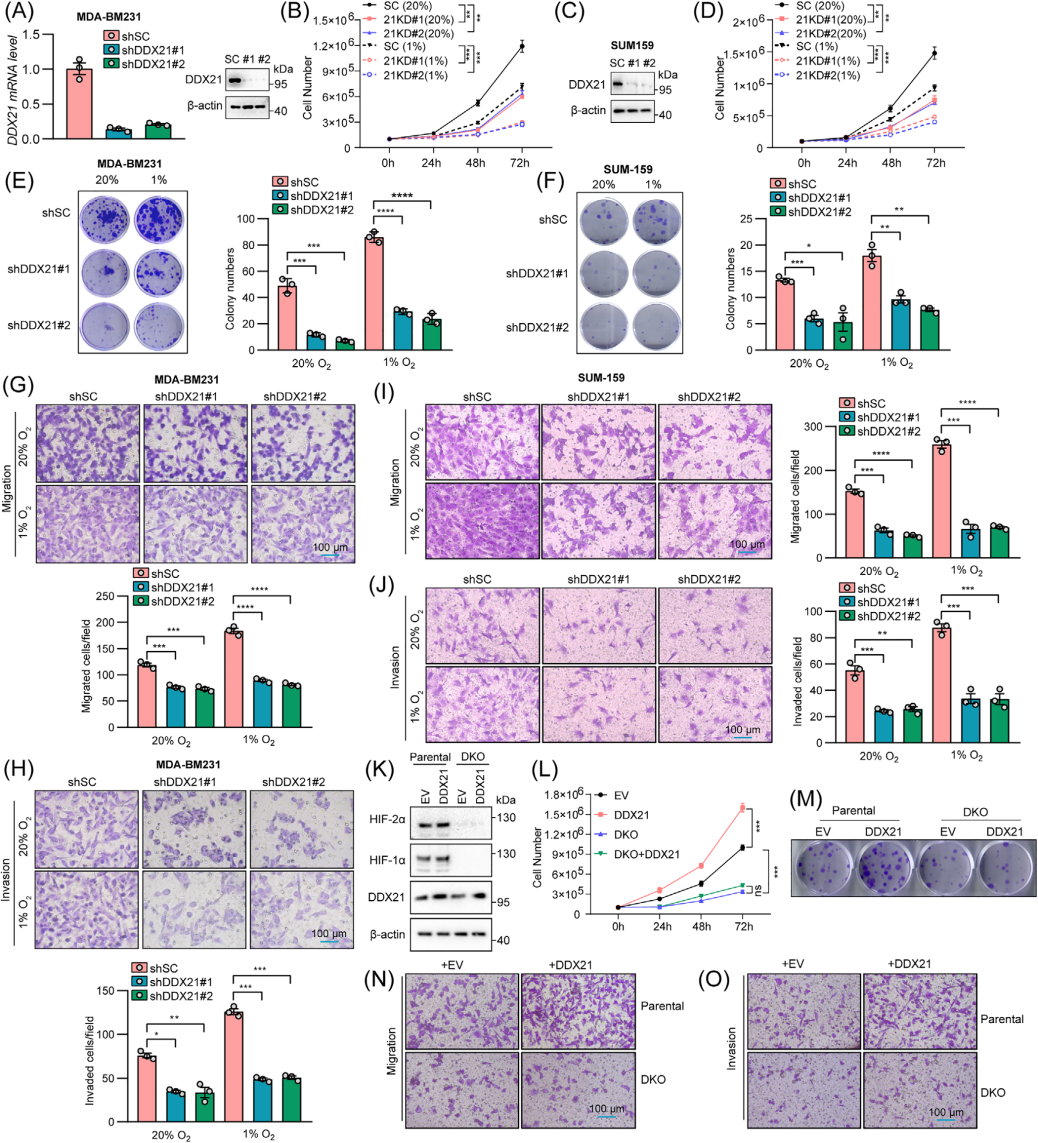
DDX21 knockdown inhibits growth and migration of breast cancer cells in vitro. (A and B) Identification of DDX21‐knockdown cell lines in MDA‐MB231 and evaluation of the effect of DDX21 knockdown on MDA‐MB231 cell proliferation (*n* = 3, mean ± SEM, ***p < 0.001). (C and D) Identification of DDX21‐knockdown cell lines in SUM‐159 and evaluation of the effect of DDX21 knockdown on SUM‐159 cell proliferation (*n* = 3, mean ± SEM, ***p < 0.001). (E and F) Clonogenic assays to evaluate the effect of DDX21 knockdown on cell survival of MDA‐MB231 (E) and SUM‐159 (F) cells (*n* = 3, mean ± SEM, *p < 0.05, **p < 0.01, ***p < 0.001, ****p < 0.0001). (G and H) Evaluate the effects of DDX21 knockdown on MDA‐MB231 cell migration and invasion (*n* = 3, mean ± SEM, *p < 0.05, **p < 0.01, ***p < 0.001, ****p < 0.0001). (I and J) Evaluate the effects of DDX21 knockdown on SUM‐159 cell migration and invasion (*n* = 3, mean ± SEM, *p < 0.05, **p < 0.01, ***p < 0.001, ****p < 0.0001). (K) Immunoblot analysis of the indicated proteins in parental and HIF‐1/2α double knockout (DKO) MDA‐MB231 cells expressing either empty vector (EV) or DDX21. (L) Evaluate the influence of DDX21 knockdown on the proliferation of MDA‐MB231 cells. (M) Colony formation assay to assess the impact of DDX21 knockdown on the survival of MDA‐MB231 cells. (N and O) Transwell assays evaluated the effect of DDX21 knockdown on MDA‐MB231 cell migration and invasion.

To further explore the oncogenic effect of DDX21 in BRCA and its association with the hypoxic microenvironment, we generated stable DDX21‑knockdown cell lines in the MDA‐MB231 (Figure 4A), SUM‐159 (Figure 4C), and MCF‐7 cell line (Figure S6A), then performed proliferation and clonogenic assays. Our findings revealed a significant inhibition of cell proliferation in MDA‐MB231 and SUM‐159 cells following DDX21 knockdown under both hypoxic and normoxic conditions (Figure 4B,D), with a similar effect observed in MCF‐7 cells (Figure S6A). The clonogenic assays showed that DDX21 knockdown significantly inhibited the clonogenicity of MDA‐MB231 (Figure 4E), SUM‐159 (Figure 4F), and MCF‐7 cells (Figure S6B) under normoxic or hypoxic conditions, indicating that DDX21 supports BRCA cell viability in hypoxia. Migration and invasion assays demonstrated that hypoxia enhanced cell migration and invasion. Notably, DDX21 knockdown significantly attenuated hypoxia enhanced cell migration and invasion in MDA‐MB231 (Figure 4G,H) and SUM‐159 cells (Figure 4I,J). Comparable results were observed when DDX21 was knocked down in MCF‐7 cells (Figure S6C,D). Moreover, DDX21 knockdown in Huh7 hepatocellular carcinoma cells also impaired tumor growth and migration (Figure S6E–G), suggesting that the protumorigenic role of DDX21 under hypoxia may extend beyond BRCA to other solid tumors.

To further determine whether DDX21 drives tumor growth and metastasis under hypoxia in an HIF‐dependent manner, we overexpressed DDX21 in parental and HIF‐knockout cells (Figure 4K) and evaluated functional outcomes. Our results indicated that overexpression of DDX21 in wild‐type cells enhanced tumor survival, migration, and invasion under hypoxia. By contrast, in HIF knockout cells, DDX21 overexpression failed to rescue the impairment in growth (Figure 4L), survival (Figure 4M), migration (Figure 4N), and invasion (Figure 4O) caused by HIF loss. Collectively, these results indicate that DDX21 promotes hypoxia‐driven tumor cell migration and invasion in an HIF‐dependent manner.

### 2.5 DDX21 Augments Breast Tumor Growth in Mice

**FIGURE 5:**
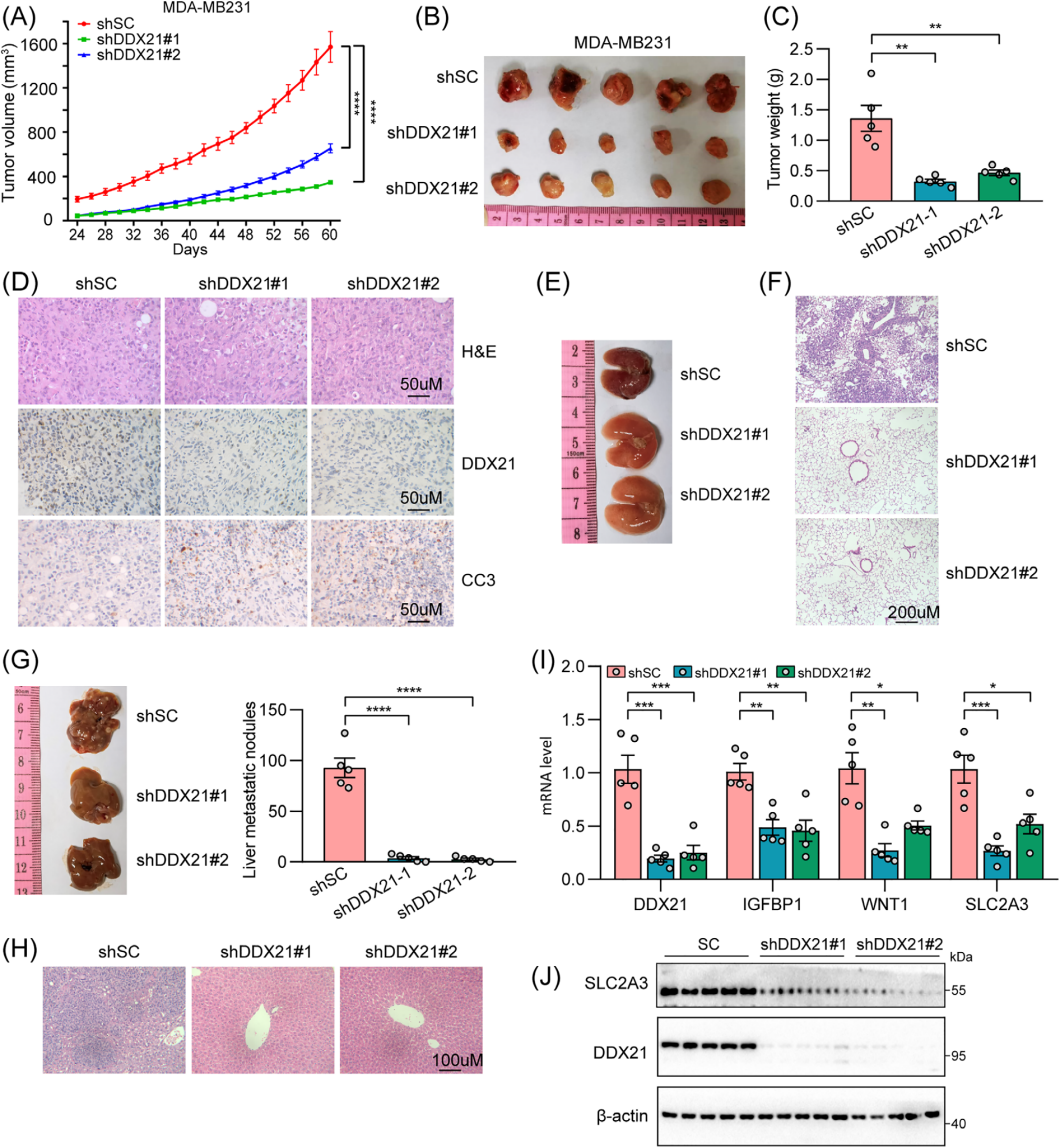
DDX21 promotes breast cancer growth and metastasis. (A–C) Growth curves (A), tumor size (B) and weight (C) of MDA‐MB231 cells (shSC) and DDX21 knockdown MDA‐MB231 cells (shDDX21#1 and shDDX21#2) (*n* = 5, mean ± SEM, **p < 0.01, ****p < 0.0001). (D) H&E pathological staining of xenograft tumors and IHC staining of tumor tissues with DDX21 and cleaved caspase‐3 (CC3). (E and F) Representative photographs of lung tissue and H&E staining of lung tissue sections. (G and H) Photographs of liver metastatic nodules and H&E pathological staining of liver tissue sections (*n* = 5, mean ± SEM, ****p < 0.0001). (I) qPCR analysis of HIF target gene expression in xenograft tumors (*n* = 5, mean ± SEM, *p < 0.05, **p < 0.01, ***p < 0.001). (J) Immunoblotting analysis of DDX21 and SLC2A3 expression in mouse xenograft tumors.

To further investigate the impact of DDX21 on BRCA growth and metastasis in vivo, we inoculated control (shSC) and DDX21‐knockdown (shDDX21#1 and shDDX21#2) cells into the mammary fat pad of female NCG mice. DDX21 knockdown significantly slowed tumor progression, as shown by the tumor growth curve (Figure 5A). Both final tumor volume and weight were markedly lower in the DDX21‑knockdown groups compared with the control group (Figure 5B,C). Consistent results were obtained in MCF‑7 xenograft tumors (Figure S7). Immunohistochemistry (IHC) assays revealed a notable increase in cleaved caspase‑3 (CC3)‑positive cells upon DDX21 knockdown (Figure 5D), indicating elevated apoptosis. Hematoxylin and eosin (H&E) staining of lung tissues showed numerous metastatic foci in control mice, whereas no evident lung metastases were detected in DDX21‑knockdown mice (Figure 5E,F). Macroscopic examination of livers revealed visible metastatic nodules in control animals, but none in the knockdown group (Figure 5G). Corresponding H&E staining confirmed that DDX21 knockdown significantly suppressed liver metastasis (Figure 5H). We further extracted tumor tissue RNA and performed qPCR to verify the effect of DDX21 on the expression of HIF target genes. The results revealed a significant reduction in the expressions of IGFBP1, WNT1, and SLC2A3 following DDX21 knockdown (Figure 5I). Consistently, protein levels of SLC2A3 were also markedly decreased (Figure 5J). Taken together, these findings demonstrate that DDX21 promotes breast tumor growth and metastasis in vivo.

### 2.6 DDX21 is a Potential Prognostic Factor in BRCA

**FIGURE 6:**
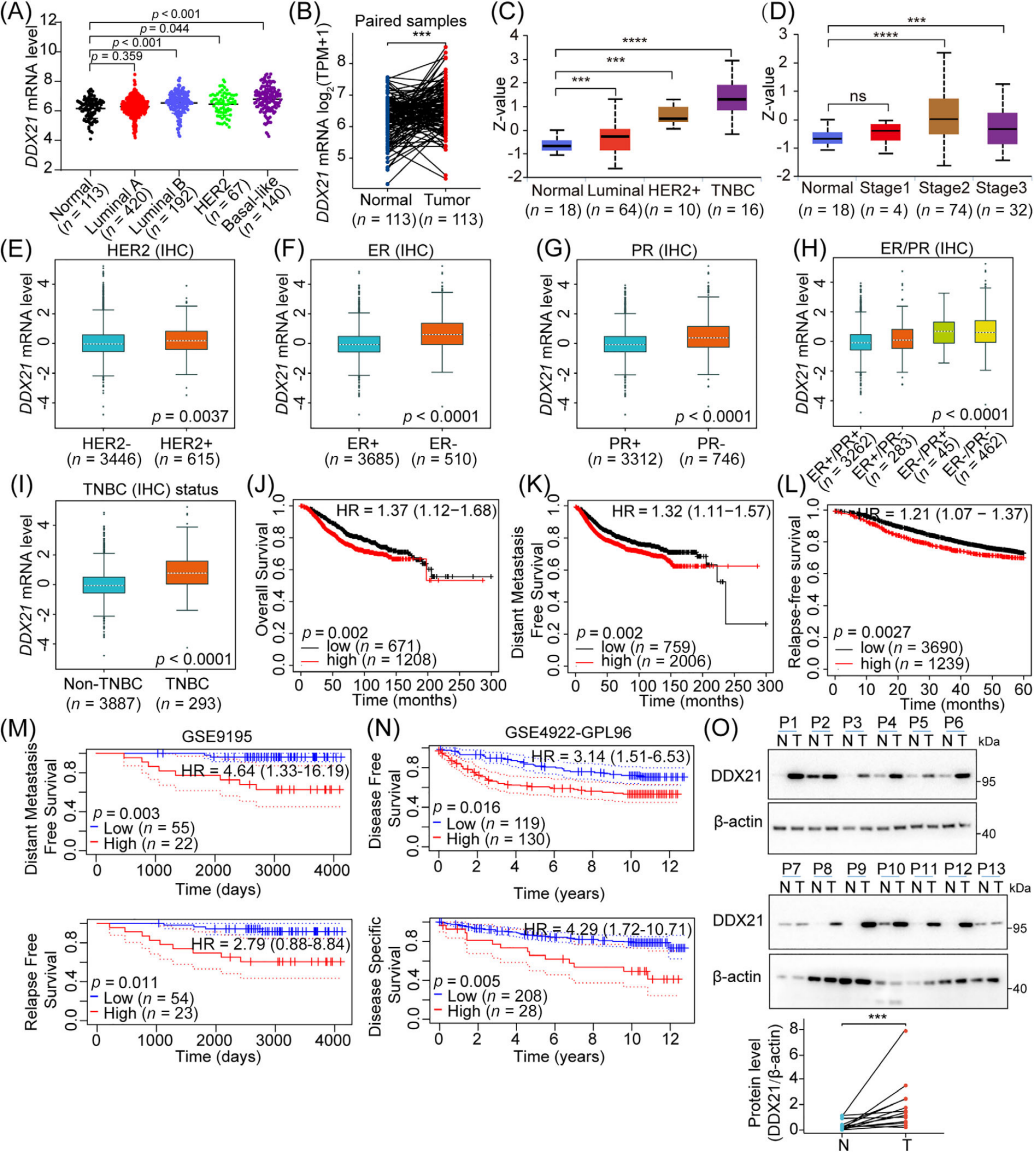
Patients with high expression of DDX21 have poorer prognosis in breast cancer (A) DDX21 mRNA expression levels across different subtypes of breast cancer in TCGA. (B) Comparison of DDX21 mRNA expression between breast cancer tissues and their paired adjacent normal tissues (***p < 0.001). (C and D) DDX21 protein expression in different breast cancer subtypes (C) or stages (D) (***p < 0.001, ****p < 0.0001). (E–I) DDX21 mRNA expression levels in breast cancer from the TCGA database, divided according to ER, PR, and HER2 expression status. (J–L) Correlation between DDX21 expression levels and survival outcomes in breast cancer patients from TCGA. (M and N) Association between DDX21 expression and clinical outcomes in the GEO database. (O) Immunoblot analysis of DDX21 protein expression in breast cancer tissues and adjacent normal tissues of patients (***p < 0.001).

To investigate the expression characteristics of DDX21 in BRCA and its clinical significance, we systematically analyzed multiple public databases and patient sample data. The results revealed that DDX21 mRNA levels were elevated in Luminal B, HER2‐enriched, and Basal‐like BRCA subtypes (Figure 6A). In‐depth analysis of 113 paired BRCA and adjacent normal tissues confirmed higher DDX21 mRNA expression in tumors (Figure 6B). Consistently, evaluation of the DDX21 protein levels in the Clinical Proteomic Tumor Analysis Consortium (CPTAC) protein database revealed increased DDX21 protein levels in BRCA tissues (Figure 6C,D). Furthermore, DDX21 mRNA levels were notably higher in epidermal growth factor receptor 2 (HER2)‐positive than in HER2‐negative patients (Figure 6E). Compared with estrogen receptor (ER) or progesterone receptor (PR)‐positive BRCA, DDX21 mRNA levels were higher in ER‐negative (Figure 6F), PR‐negative (Figure 6G), and ER−/PR− (Figure 6H) BRCA. Additionally, DDX21 expression in triple‐negative BRCA (TNBC) was significantly elevated compared with non‐TNBC (Figure 6I). In essence, DDX21 exhibited a more pronounced increase in more aggressive BRCAs, including HER2‐positive, ER−/PR−, and triple‐negative BRCAs. Beyond BRCA, DDX21 mRNA and protein levels were also significantly elevated in multiple other cancers, such as colorectal cancer, lung cancer, and hepatocellular carcinoma (Figure S8A,B). Furthermore, BRCA patients with high expression of DDX21 exhibited poor overall survival (OS), distant metastasis‐free survival, and recurrence‐free survival (Figure 6J–L). Consistent with these findings, analysis of GEO database also suggested elevated DDX21 expression led to shorter survival times (GSE9195, GSE4922–GPL96, GSE3494–GPL96, GSE12276, GSE9893) (Figures 6M,N and S8). Additionally, we examined the expression of DDX21 protein in BRCA tissues and adjacent normal tissues of patients, and the results confirmed higher DDX21 levels in cancerous tissues (Figure 6O). Similarly, in liver hepatocellular carcinoma, patients with high DDX21 expression exhibited significantly shorter OS, disease‐specific survival, and progression‑free interval (Figure S8F). Collectively, these results indicate that elevated DDX21 expression is significantly correlated with a poorer prognosis in both human BRCA and hepatocellular carcinoma.

## 3 Discussion

Hypoxia is a defining hallmark of most solid tumors, although its occurrence and severity vary among different patient cohorts. Hypoxia arises because of oxygen diffusion limitations in avascular primary tumors or their metastases [44]. Due to its contributions to multiple facets such as chemotherapy/radiotherapy resistance, angiogenesis, invasiveness, metastasis, resistance to cell death, metabolic alterations, and genomic instability, hypoxia represents a negative prognostic and predictive factor [45]. The transcriptional activity of HIF is not autonomously determined but is precisely controlled by a sophisticated network of coregulators [46]. The binding of HIF to the DNA of its target genes is merely the initial step in transcription initiation; subsequent robust activation relies on HIF functioning as a molecular scaffold to recruit a diverse array of coregulators, including p300/CBP [16, 17], TIP60 complex [18], chromatin remodeling factors (JMJD2C [19], JMJD1A [20], and TET1 [47], SET9 [48], SET1B [49]), and epigenetic enzymes (PADI4 [22], ZMYND8 [40, 50], CHD4 [51]), CDK8‐Mediator [39], and TRIM28 [52]. These coregulators act synergistically to modulate the process at various levels, such as chromatin opening and the recruitment of the transcriptional machinery, thereby collectively determining the specificity, potency, and duration of HIF's transcriptional output. Within this complex network, transcriptional elongation represents a rate‐limiting step for efficient gene expression. Although P‑TEFb activity is modulated by complexes like SEC and BRD4 [39, 40], it remains unclear how the inhibitory 7SK RNP complex is regulated and participates in HIF‐mediated transcriptional elongation during hypoxia.

Here, we found that DDX21 is upregulated in BRCA and that high DDX21 expression correlates with poor prognosis. DDX21 promoted BRCA tumor growth and metastasis in vitro and in vivo. Mechanistically, HIF binds to DDX21, recruiting it to specific target genes of HIFs. Subsequently, DDX21 facilitates dissociation of CDK9 from the 7SK complex, thereby enhancing P‑TEFb‑mediated elongation of HIF target genes under hypoxia. Transcriptome sequencing revealed that DDX21 regulates a set of HIF‑specific target genes, including *PCK1, IGFBP1, SLC2A3, WNT1, WNT10B*, and others. Previous works have revealed that these genes, as HIF target genes, play a vital role in tumor progression. Specifically, hypoxia triggers the expression of *WNT1* and *WNT10B*, which facilitate cell cycle re‐entry [53]. IGFBP1, identified as a HIF target gene [54], is known to regulate tumor behaviors such as proliferation, migration, invasion, and adhesion [55]. Furthermore, SLC2A3, also known as GLUT3, serves as a hypoxia marker [56] and acts as a tumor promoter and accelerates aerobic glycolysis [57]. Additionally, research indicates that hypoxia induces the expression of PCK1, a gluconeogenic enzyme induced by hypoxia, which stimulates the growth of hypoxic tumor‑repopulating cells through metabolic reprogramming [58]. Our work thus identifies the DDX21–7SK axis as a previously unrecognized layer of regulation that, alongside the SEC and BRD4 complexes, fine‑tunes P‑TEFb activity in a subset of HIF‑driven transcriptional programs essential for breast‑cancer growth and metastasis. The DDX21–7SK axis does not mediate all HIF‑dependent transcription, but together with other coregulators it forms an intricate network that shapes the specificity of the hypoxic transcriptional response.

DDX21 is composed of an N‐terminal domain, a helicase core domain (consisting of D1 and D2 subdomains), and a C‐terminal foldase core domain [25, 59]. Previous studies demonstrated that both DDX21 SAT (a catalytic‐deficient mutant) and DDX21 DEV fail to release P‐TEFb from inactive 7SK snRNP complexes, as these functional sites reside within the DEAD domain [36]. This indicates that DDX21 requires its intact helicase domain to drive Pol II‐dependent transcription by releasing P‐TEFb. Our findings reveal three independent DDX21 domains capable of binding HIFs: DEAD domain, Helicase domain (HeliC), and C‐terminal domain. Whether these domains act synergistically or redundantly in regulating P‐TEFb release under hypoxia remains unclear. Structural elucidation of the DDX21–HIF interface would facilitate the rational design of DDX21 inhibitors.

There are three HIF‐α isoforms: HIF‐1α, HIF‐2α, and HIF‐3α. They form heterodimeric transcription factors with the common HIF‐1β subunit, known as HIF‐1, HIF‐2, and HIF‐3, respectively [60, 61]. All HIF‑α isoforms share a bHLH motif that mediates DNA binding, an oxygen‐dependent degradation domain (ODDD) responsible for proline hydroxylation and lysine to trigger VHL‐mediated proteasomal degradation, an N‐terminal transcriptional activation domain (N‐TAD) embedded within the ODDD for precise transcriptional regulation, and a C‐terminal transcriptional activation domain (C‐TAD) that binds to coactivators such as p300/CBP to drive gene transcription [62]. HIF‑3α, however, gives rise to multiple splice variants; some lack the C‑TAD and may act as competitive inhibitors of HIF‑1/2α, while others contain an LZIP domain involved in DNA binding and protein–protein interactions [63, 64]. HIF‐1 dominates acute hypoxic responses (e.g., angiogenesis and glycolysis), while HIF‐2 regulates long‐term adaptations (e.g., erythropoiesis and vascular remodeling). These two isoforms typically function synergistically and play a central role in oxygen homeostasis and cancer metabolism [62]. In contrast, HIF‐3 remains less studied, and whether it antagonizes or cooperates with HIF‐1α/HIF‐2α requires further investigation. Our study demonstrates that DDX21 can bind to both HIF‐1α and HIF‐2α. Given the high homology among HIF‐α subunits, DDX21 may also interact with HIF‐3α, but the functional consequences of such an interaction are unpredictable owing to the pleiotropic and poorly defined roles of HIF‑3.

**FIGURE 7:**
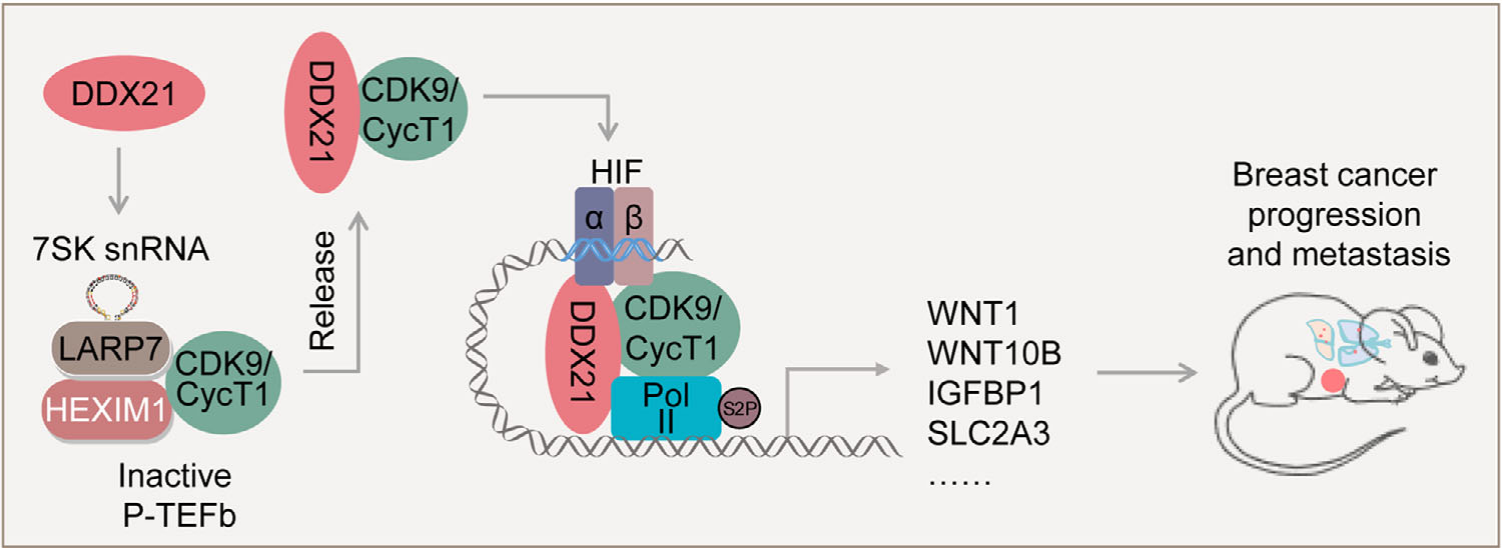
Proposed model of DDX21 in regulation of HIF transcriptional activity and breast cancer progression. DDX21 promotes CDK9 release from the 7SK complex, enhancing CDK9 interaction with HIFα and Pol II. This leads to increased Pol II‐S2P recruitment to HREs of HIF target genes, stimulating their transcription elongation, supporting hypoxic adaptation, and ultimately fostering tumor growth and metastasis.

Taken together, our research has uncovered that DDX21 induces the release of CDK9 from the 7SK complex, enhancing the interaction between CDK9 and HIF‐1α. This interaction stimulates the transcription elongation of HIF target genes by elevating the enrichment of phosphorylated RNA Pol II at serine 2 (Pol II‐S2P) on the HRE of HIF target genes, stimulating their transcriptional elongation and thereby enabling tumor cells to adapt to hypoxic stress, which ultimately drives tumor growth and metastasis (Figure 7). Despite these advances, several limitations remain in terms of mechanistic depth, physiological relevance, and translational application. First, the undefined binding interface and structure of the DDX21–HIF complex limit mechanistic insight and rational drug design, necessitating structural studies and mutational analysis. Second, conventional immunodeficient xenograft models lack physiological relevance due to inadequate immune microenvironment representation; tissue‐specific knockout models or patient‐derived organoids are required for more accurate functional evaluation. Finally, the unverified therapeutic potential calls for DDX21‐based screening platforms to enable high‐throughput inhibitor discovery and efficacy validation, bridging target identification and interventional confirmation.

## 4 Materials (or Subjects) and Methods

### 4.1 Cell Lines

HEK293T, MDA‐MB231, MCF‐7, T47D, Huh7, and HeLa cells were cultured in Dulbecco's modified Eagle's medium supplemented with 10% fetal bovine serum (FBS). SUM‐159 cells were maintained in RPMI‐1640 medium with 10% FBS. All cells were maintained at 37°C in a 5% CO_2_ and 95% air incubator (20% O_2_). Hypoxic cells were cultured in modular incubator chamber (Billups‐Rothenberg) flushed a gas mixture of 1% O_2_/5% CO_2_/94% N_2._

### 4.2 Animals

Severe immune‐deficient strain NCG (NOD/ShiLtJGpt‐Prkdc^em26Cd52^Il2rg^em26Cd22^/Gpt) mice were purchased from the GemPharmatech Co., Ltd (Guangdong, China). The animals were raised and kept in specific pathogen‐free environment at the Experimental Animal Center of the Jinan University (Guangzhou, China). The experimental protocols gained approval from the Institutional Animal Care & Use Committee (IACUC) guidelines of Jinan University. Approval number for animal: IACUC‐20220331‐12.

### 4.3 Human Subjects

A total of 13 tumor specimens and 13 adjacent tissues from patients with pathological diagnosis of BRCA were enrolled at the Department of Oncology, First Affiliated Hospital of Gannan Medical University. Our study protocols gained approval from the Ethics Committees of the First Affiliated Hospital of the Gannan Medical University, and every human experiment was conducted in accordance with the guidelines of the Declaration of Helsinki. Each participant offered their written informed consent. Approval number for clinical experiment: LLSC‐2023‐534.

### 4.4 Plasmid Constructs

The annealed gRNA we cloned into BsmBI‐digested lentiCRISPR v2 vector. shRNA oligos was cloned into the pLKO.1‐puro vector digested with *Age*I and *Eco*RI. The full length of DDX21 and various domains were amplified by PCR and then cloned into pCMV‐HA vector. The promoters of IGFBP1 and WNT1 were cloned into the PGL3‐basic vector. All the primers were listed in the Tables S2–S4.

### 4.5 Lentivirus Transduction

For lentiviral production, HEK293T cells were co‐transfected with the packaging plasmids psPAX2 and pMD2.G alongside either LentiCRISPR v2 constructs targeting HIF‐1αor HIF‐2α, pLKO.1‐shDDX21, or PCDH‐DDX21.. Viruses were harvested 48 h after transfection and passed through a 0.45 µm filter. Puromycin was added to the culture medium of lentiviral transduced cells to select stable cells at a concentration of 0.5–1 mg/mL.

### 4.6 Quantitative Polymerase Chain Reaction

Total RNA was isolated from cultured cells or tissues using Trizol reagent and reverse transcribed using PrimeScript RT Master Mix kit. RT‐qPCR assays were conducted utilizing the primers listed in the Table S1.

### 4.7 RNA‐seq Assay

RNA extraction, RNA quality assessment, RNA library construction, sequencing, and data analysis were conducted by BGI (Beijing Genomics Institute). The raw sequence data reported in this paper have been deposited in the Genome Sequence Archive in National Genomics Data Center, China National Center for Bioinformation/Beijing Institute of Genomics, Chinese Academy of Sciences (GSA‐Human: HRA010676) that are publicly accessible at https://ngdc.cncb.ac.cn/gsa‐human.

### 4.8 Protein Purification and Pull‐Down Assay

pT7‐GST‐HIF‐1α, pT7‐GST‐HIF‐2α, and pT7‐6×His‐DDX21 were separately transformed into BL21 competent E. coli and induced with IPTG to express the fusion proteins. After induction, bacterial lysates were prepared, and the GST‐fusion proteins were directly incubated with Glutathione‑Sepharose 4B beads (Cytiva; 17‑0756‑01) and washed to remove nonspecific binders, while the His‑fusion proteins were purified using HisSep Ni‑NTA MagBeads (Yeasen; 20561ES08). For the interaction assay, purified His‑DDX21 was then added to Glutathione‑Sepharose 4B beads that had been preloaded with the GST‑fusion proteins to allow specific interaction. The beads were subsequently washed five times with lysis buffer to remove unbound proteins, and the bound complexes were eluted by boiling in sodium dodecyl sulfate‐polyacrylamide gel electrophoresis (SDS‑PAGE) loading buffer. Samples were analyzed by SDS‑PAGE followed by Western blotting to detect His‑DDX21 associated with the GST moiety, thereby verifying whether a direct or indirect interaction occurred between the two partners.

### 4.9 Immunoprecipitation

Cells were lysed in IP buffer (25 mM Tris–HCl [pH 7.6], 150 mM NaCl, 0.5% NP‐40, 0.5% Triton X‐100, 10% glycerol) supplemented with protease inhibitors. Protein concentrations were determined using a BCA Protein Assay Kit. For IP experiments, antitarget protein antibody, or control IgG (for negative control) was coupled to protein A magnetic beads following the manufacturer's instructions. The cell lysates were incubated with the antibody‐coupled beads at 4°C overnight with gentle agitation. Following the incubation, the beads were washed extensively with IP buffer, and the bound protein complexes were eluted for subsequent analysis.

### 4.10 Immunoblot Assays

Proteins from the IP or total cell lysates were separated by SDS‐PAGE and transferred onto a polyvinylidene fluoride membrane. The membrane was blocked in 5% non‐fat milk in TBST (Tris‐buffered saline with Tween 20) for 1 h at room temperature. Subsequently, the membrane was probed with specific primary antibodies against at the appropriate dilutions. After overnight incubation at 4°C, the membrane was washed and incubated with corresponding HRP‐conjugated secondary antibodies. Protein bands were visualized using an enhanced chemiluminescence detection system.

### 4.11 Nuclear Protein and Cytoplasmic Protein Extraction

Nuclear protein and cytoplasmic protein were extracted by using the Nuclear Extraction Kit (Beyotime; P0027). First, cells were washed with PBS, scraped, and centrifuged to get the pellet. Then, PMSF‐containing cell lysis buffer A was added, vortexed to suspend cells, incubated on ice for 10–15 min, followed by adding buffer B, vortexing again, and incubating on ice for 1 min. The mixture was centrifuged at 12,000×*g*, 4°C for 5 min, and the supernatant was collected as cytoplasmic protein. Next, the pellet was resuspended in cold PBS, re‐centrifuged, and PMSF‐containing nuclear protein extraction buffer was added. After vortexing for 15–30 s, it was incubated on ice with vortexing every 5 for 30 min. Finally, the mixture was centrifuged at 12,000×*g*, 4°C for 10 min, and the supernatant was collected as nuclear protein.

### 4.12 Luciferase Reporter Assay

HEK293T or HeLa cells were seeded at a density of 5 × 10^4^ cells per well in a 48‐well plate and then transfected with indicated plasmids and subsequently exposed to 20 or 1% O_2_ for 24 h. Cell lysates were collected for Luciferase activity measurement using the Dual Luciferase Reporter Assay System (Promega), with Renilla luciferase serving as the normalization control.

### 4.13 Chromatin Immunoprecipitation Assay

Cells were harvested and cross‐linked with 1% formaldehyde, then quenched the cross‐linking reaction with 0.125 M glycine. Chromatin was isolated using the Pierce Magnetic ChIP Kit (Pierce), sonicated to 200–300 bp in length, and subjected to IP using antibodies against S2P. ChIP DNA were prepared for quantitative PCR with the primers listed in Table S5.

### 4.14 CUT&RUN Assay

Using the Hyperactive pG‐MNase CUT&RUN Assay Kit for Illumina (Vazyme; HD102) and following the standard protocol, the experimental procedure is briefly outlined as follows: first, the necessary buffers, such as binding buffer and wash buffer, were prepared according to the single‑sample proportions (note that Digitonin was freshly prepared before use). Subsequently, ConA beads were equilibrated with binding buffer and then incubated with cells at room temperature for 10 min to allow cell adsorption onto the beads. Next, prechilled Antibody Buffer and the target protein primary antibody (RNA Pol II‑S2P) were added, followed by incubation at room temperature for 2 h. This was followed by the addition of diluted pG‑MNase enzyme, which was incubated with the cell‑bead complexes at 4°C with rotation for 1 h to enable targeted binding to the DNA–protein interaction sites. Calcium chloride was then added to activate MNase, initiating a 90‑min cleavage reaction on ice to digest the target DNA. The reaction was terminated with stop buffer, and DNA fragments were released by static incubation at 37°C, followed by purification using spin columns. Finally, the extracted DNA was subjected to end repair, adapter ligation, and PCR amplification to construct the sequencing library. The library was subjected to quality control and sequencing by Annoroad Gene Technology, and subsequent data analysis was performed on the Vazyme cloud platform (http://cloud.vazyme.com:83/).

### 4.15 Clonogenic Assay

A total of 200 tumor cells (MDA‐MB231, SUM‐159, or Huh7) were seeded into 12‐well plates and cultured under hypoxic and normoxic conditions for 14 days. For MCF‐7 cells, 100 cells were seeded into 12‐well plates and cultured under identical experimental conditions with parallel hypoxia and normoxia exposure. Colonies were washed with PBS, fixed with 4% paraformaldehyde (PFA) for 20 min, and stained with 0.5% crystal violet for 20 min. After staining, colonies were scanned or photographed.

### 4.16 Migration and Invasion Assays

A total of 10^5^ tumor cells were resuspended in serum‐free medium and seeded in the transwell insert (migration) to ensure a serum‐free environment, for invasion assays, the matrigel should be coated on transwell insert beforehand. Then, complete medium with 10% FBS was added to the bottom chamber. The cells were exposed to 20 or 1% O_2_ for 16–24 h. Cells were fixed with methanol, stained with 0.5% crystal violet, and photographed under the microscope. Cells that migrated to the underside of the polycarbonate membrane were counted.

### 4.17 Tumor Xenograft Model

Xenograft assay was performed as previously described [65]. In brief, 1.5 × 10^6^ MDA‐MB231 or MCF‐7 cells were resuspended in 100 µL sterile PBS/Matrigel (1:1; Corning) and then injected into the mammary fat pad. For MCF‐7 group, 100 µL of 10 mM estradiol was topically administered to the neck of each mouse every other day, starting 1 day post MCF‐7 cell inoculation, until the end of the experiment. Tumor growth was monitored by measuring tumor dimensions with calipers every 2 days beginning on Day 24 (MDA‐MB231) or Day 32 (MCF‐7) after cell implantation, and tumor volume was calculated using the formula *V* = (length × width^2^)/2. Mice were euthanized, and tumors were excised at the end of the study or when ethical endpoint criteria were met. Tumor, liver, and lung tissues were fixed, sectioned, and subjected to H&E staining and IHC for evaluating tumor histology.

### 4.18 Histological Analysis

Mouse tumors and organs procured, fixed in 4% PFA and dehydrated through a gradient of ethanol concentrations. The samples were embedded in paraffin and sectioned into 5‐micron thick slices. After rehydration, the sections were stained with H&E. For IHC, sections were incubated overnight with anti‐DDX21 (1:500) and anticleaved‐caspase‐3 (1:500) antibodies. Following incubation, the sections were washed with PBS, then incubated with a secondary antibody. Staining was visualized with DAB, and nuclei were counterstained with hematoxylin. The stained sections were then observed and photographed under a microscope.

### 4.19 Data Acquisition from Online Database

The mRNA expression data of DDX21 were obtained from the TCGA BRCA cohort (https://www.aclbi.com/static/index.html#/tcga). Expression of DDX21 across BRCA molecular subtypes (defined by IHC‑based ER/PR/HER2 status) was analyzed through the BC‑GenExMiner platform (https://bcgenex.ico.unicancer.fr/BC‐GEM/GEM‐Accueil.php?js=1), integrating transcriptomic data from 4421 patients (TCGA cohort: *n* = 743; SCAN‑B cohort: *n* = 3678) [66]. DDX21 protein expression analysis was performed based on The University of ALabama at Birmingham CANcer (UALCAN) data analysis Portal [67]. Hypoxia‐related genes: HARRIS_HYPOXIA gene set [41], BUFFA_HYPOXIA_METAGENE gene set [68], and WINTER_HYPOXIA_UP gene set [69] (Table S6) were searched from Molecular Signatures Database (MsigDB; http://www.gsea‐msigdb.org/gsea/index.jsp). The Pearson correlation test was used to compare expression of DDX21 with the HIF signature. Kaplan–Meier analysis was performed using an online tool (https://www.kmplot.com) [70]. Cancer single‐cell state atlas (CancerSEA) database was utilized to explore the correlation between DDX21 expression and biological processes in BRCA cells at single‐cell resolution [71].

### 4.20 Statistical Analysis

GraphPad Prism 8.0 (GraphPad Software) was employed for statistical analyses. Differences between two groups were analyzed by Student's *t* test; differences between multiple groups were analyzed by one‐way ANOVA. For correlation analysis, linear regression analysis was performed. The sample size was not predetermined by any statistical means. All results were represented by mean ± SEM, unless specially annotated in figure legends. *p < 0.05, **p < 0.01, ***p < 0.001, ****p < 0.0001, ns: not significant.

## Author Contributions

G.L., M.D., L.Z., and Z.L. carried out experiments, analyzed data, and wrote the manuscript. R.G., X.L., and X.X. analyzed data and edited the manuscript. R.C. performed clinical sample collection and assays. Q.W. assisted with animal experiments. Z.Y., Y.C., and Y.D., mentored and supervised this research. All authors have read and approved the final manuscript.

## Ethics Statement

The study was performed in accordance with the Declaration of Helsinki. All of the authors agree to the submission and consent for publication this paper. The research was approved by the Ethics Committee of Jinan University (approval number for animal: IACUC‐20220331‐12) and the First Affiliated Hospital of Gannan Medical University (approval number for clinical experiment: LLSC‐2023‐534).

## Conflicts of Interest

The authors declare no conflicts of interest.

## Supporting information




**Figure S1**: Validation of protein‐protein interactions between HIF‐1α and candidate binding partners.
**Figure S2**: DDX21 interacts with HIF‐1α and HIF‐2α.
**Figure S3**: DDX21 promotes transcription of HIF specific target genes
**Figure S4**: DDX21 promotes CDK9 binding to HIF‐1α.
**Figure S5**: Correlation between DDX21 expression and the hypoxia signature, biological processes.
**Figure S6**: DDX21 promotes the growth and migration of breast cancer and hepatocellular carcinoma cells in vitro.
**Figure S7**: DDX21 promotes breast cancer growth.
**Figure S8**: Patients with high expression of DDX21 have poorer prognosis.
**Table S1**: Primers for qPCR analysis.
**Table S2**: Primers for DDX21 FL and domain cloning.
**Table S3**: Primers for promoter cloning.
**Table S4**: shRNA or sgRNA oligos.
**Table S5**: Ch‐IP qPCR Primers.
**Table S6**: Hypoxia signatures according MsigDB.
**Table S7**: Key resources table.

## Data Availability

The data generated or analyzed during the current study are available from the corresponding author upon reasonable request.
