# Supplementary material for: DDX21 Promotes Breast Cancer Growth and Metastasis via Stimulating RNAPII Elongation During Hypoxia
Source: MedComm (2020). 2026 Jun 15;7(7):e70792. doi: 10.1002/mco2.70792 (PMC13269829; doi:10.1002/mco2.70792)
Supplement: Supplementary file 1 — Figure S1: Validation of protein‐protein interactions between HIF‐1α and candidate binding partners. Figure S2: DDX21 interacts with HIF‐1α and HIF‐2α. Figure S3: DDX21 promotes transcription of HIF specific target genes Figure S4: DDX21 promotes CDK9 binding to HIF‐1α. Figure S5: Correlation between DDX21 expression and the hypoxia signature, biological processes. Figure S6: DDX21 promotes the growth and migration of breast cancer and hepatocellular carcinoma cells in vitro. Figure S7: DDX21 promotes breast cancer growth. Figure S8: Patients with high expression of DDX21 have poorer prognosis. Table S1: Primers for qPCR analysis. Table S2: Primers for DDX21 FL and domain cloning. Table S3: Primers for promoter cloning. Table S4: shRNA or sgRNA oligos. Table S5: Ch‐IP qPCR Primers. Table S6: Hypoxia signatures according MsigDB. Table S7: Key resources table. [file MCO2-7-e70792-s001.docx]

**Supplemental information**

**DDX21 Promotes Breast Cancer Growth and Metastasis via Stimulating RNAPII Elongation during Hypoxia**

Guangqiang Li^1,2,#^, Mingxia Deng^2,#^, Leqing Zhu^2,#^, Zhiwei Lei^3,#^, Rong Guo^4,#^, Xiong Liu^5^, Renwang Chen^4^, Xichun Xia^6^, Qiong Wen^1^, Yuanyuan Duan^7,*^, Yan Chen^8,*^, Zhinan Yin^1,9,*^

^1^ The Biomedical Translational Research Institute, Health Science Center (School of Medicine), Jinan University, Guangzhou, 510632, China.

^2^ Guangzhou National Laboratory, Guangzhou International BioIsland, Guangzhou, 510005, China.

^3^ Department of Gastroenterology, Affiliated Qingyuan Hospital, Guangzhou Medical University, Qingyuan People's Hospital, Qingyuan, 511500, China.

^4^ The First Affiliated Hospital of Gannan Medical University, Ganzhou, 341000, China,

^5^ Clinical Laboratory, Hunan Aerospace Hospital, 189 Fenglin 3rd Road, Yuelu District, Changsha, 410205, China.

^6^ Institute of Dermatology and Venereal Diseases, Dermatology Hospital, Southern Medical University, Guangzhou, 510091, China.

^7^ Key Laboratory of Viral Pathogenesis & Infection Prevention and Control (Jinan University), Ministry of Education, School of Medicine, Jinan University, Guangzhou, 510632, China.

^8^ Center for Cell Structure and Function, Institute of Biomedical Science, College of Life Sciences, Shandong Normal University, Jinan, 250014, China.

^9^ The Affiliated Guangdong Second Provincial General Hospital of Jinan University, Guangzhou, 510317, China.

^#^These authors contributed equally.

^*^Correspondence:

tzhinan@jnu.edu.cn.

yanchen@sdnu.edu.cn.

yyduan@jnu.edu.cn.

**^Supplemental Figures^**


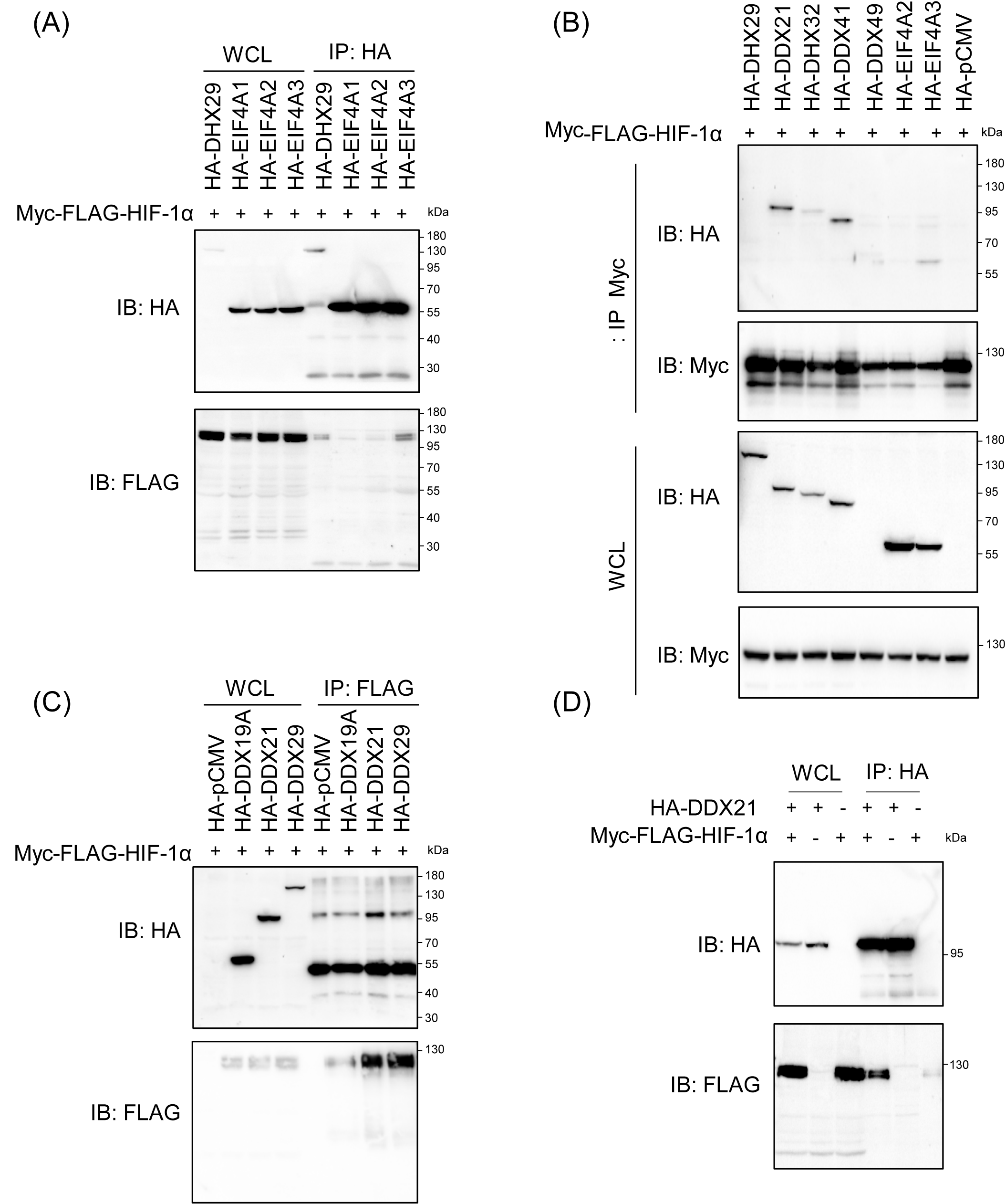


**Figure S1. Validation of protein-protein interactions between HIF-1α and candidate binding partners**

(A) co-IP analysis of Myc-FLAG-HIF-1α with HA-tagged proteins using anti-HA antibody.

(B) co-IP analysis of Myc-FLAG-HIF-1α with HA-tagged proteins using anti-Myc antibody.

(C) co-IP analysis of Myc-FLAG-HIF-1α with HA-tagged proteins using anti-FLAG antibody.

(D) Specific interaction between HA-DDX21 and Myc-FLAG-HIF-1α. Co-IP was performed using both anti-HA antibody.

**Figure S2. DDX21 interacts with HIF-1α and HIF-2α.**


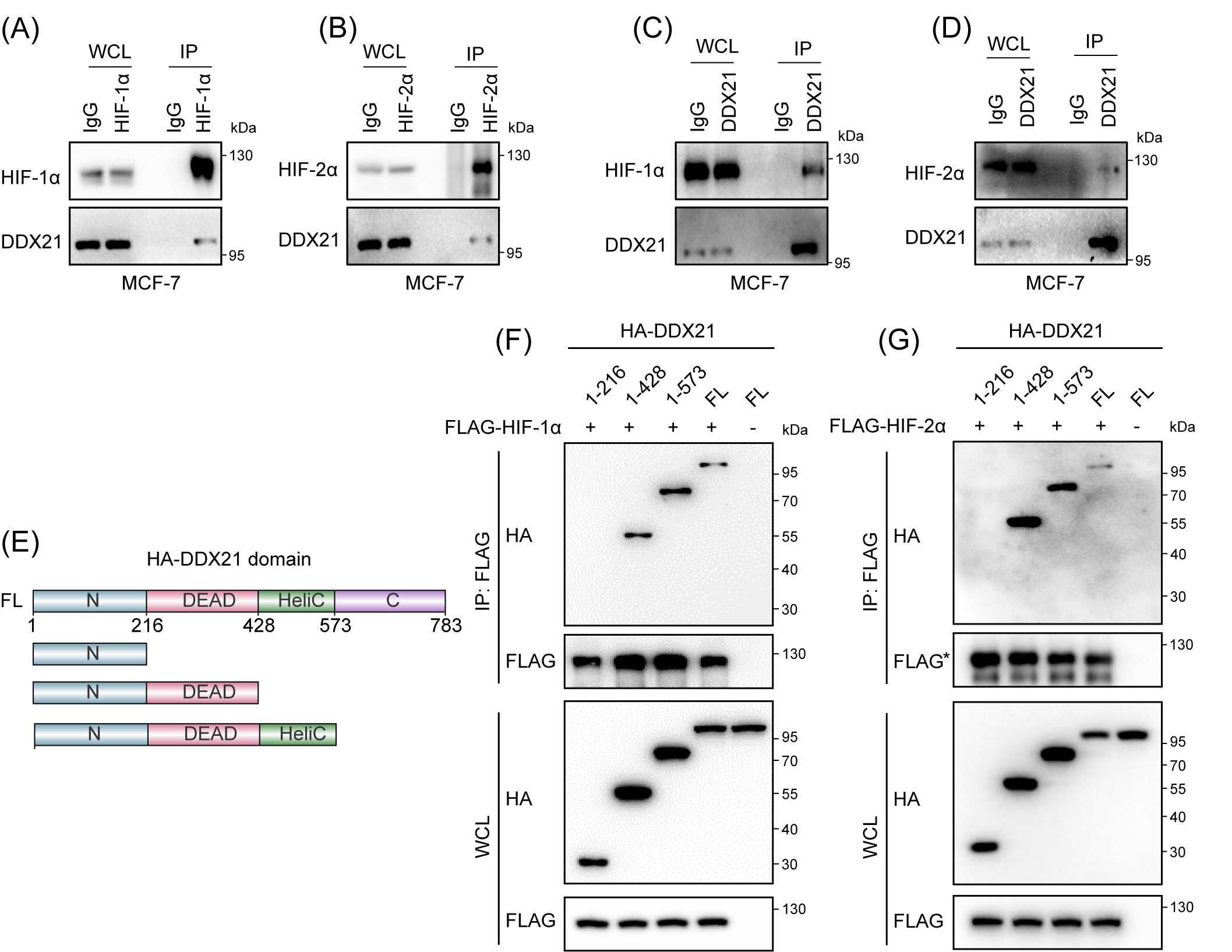


(A-D) Co-IP assays of endogenous DDX21 and HIF-1α or HIF-2α with anti-DDX21 (A, B), anti-HIF-1α (C) or anti-HIF-2α (D) antibody in MCF-7 cells exposed to 1% O_2_ for 6 hours. (E) Schematic diagram of DDX21 domain.

(F-G) Domain mapping of DDX21 binding to HIF-1α (F) and HIF-2α (G). WCL, whole cell lysate. (* indicates the target FLAG band)


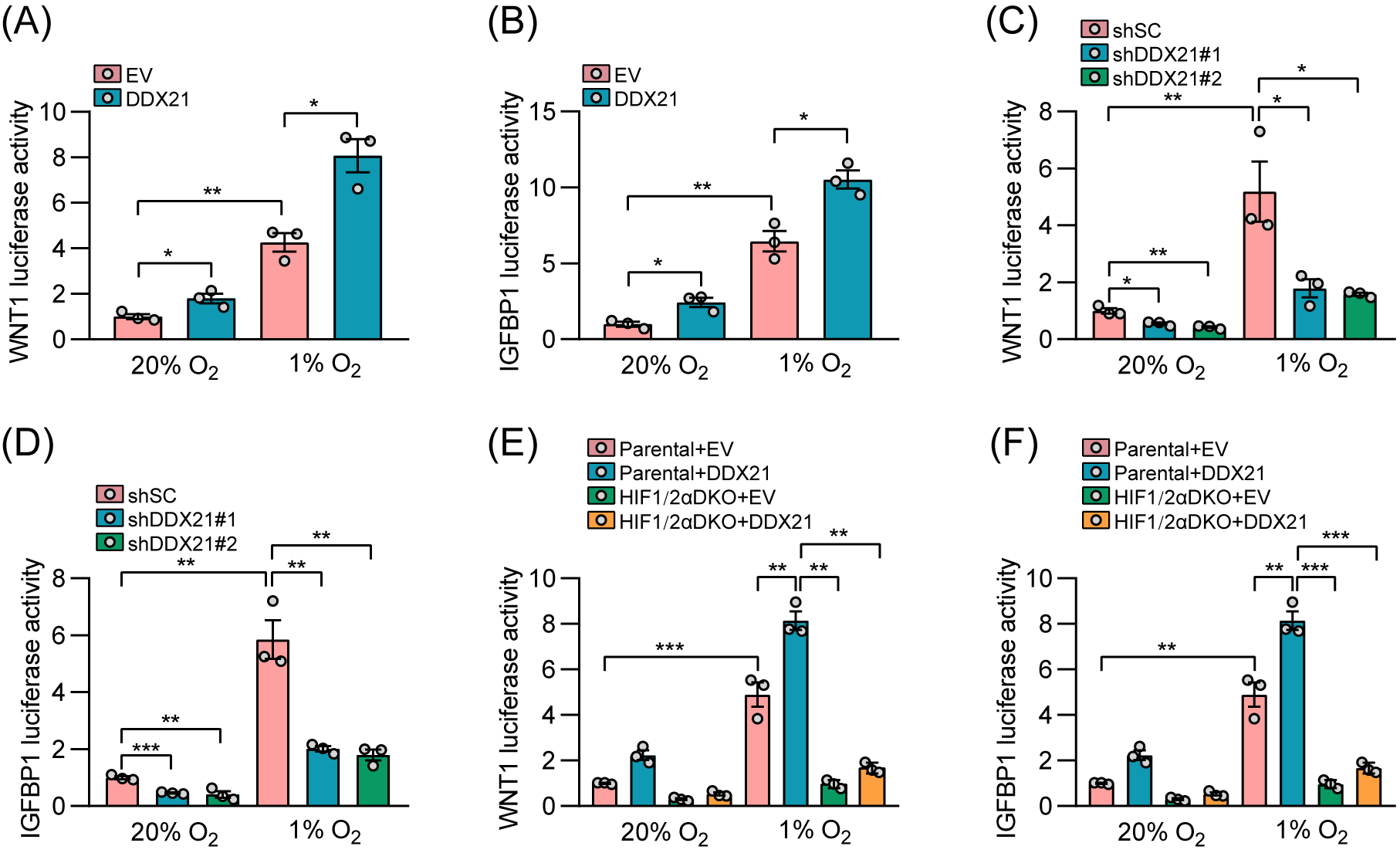


**Figure S3. DDX21 promotes transcription of HIF specific target genes**

(A-B) Overexpression of DDX21 in HEK293T cells was performed to assess its effect on HIF transcriptional activity via a dual-luciferase reporter assay. (n=3, mean ± SEM, *P<0.05, **P<0.01)

(C-D) DDX21 knockdown using shRNA was employed to investigate its impact on the transcriptional activity of HIFs. (n=3, mean ± SEM, *P<0.05, **P<0.01, ***P<0.001)

(E-F) DDX21 was overexpressed in HIF-1α/2α double-knockout (DKO) HeLa cells to examine its effect on the transcriptional activity of HIFs. (n=3, mean ± SEM, **P<0.01, ***P<0.001)


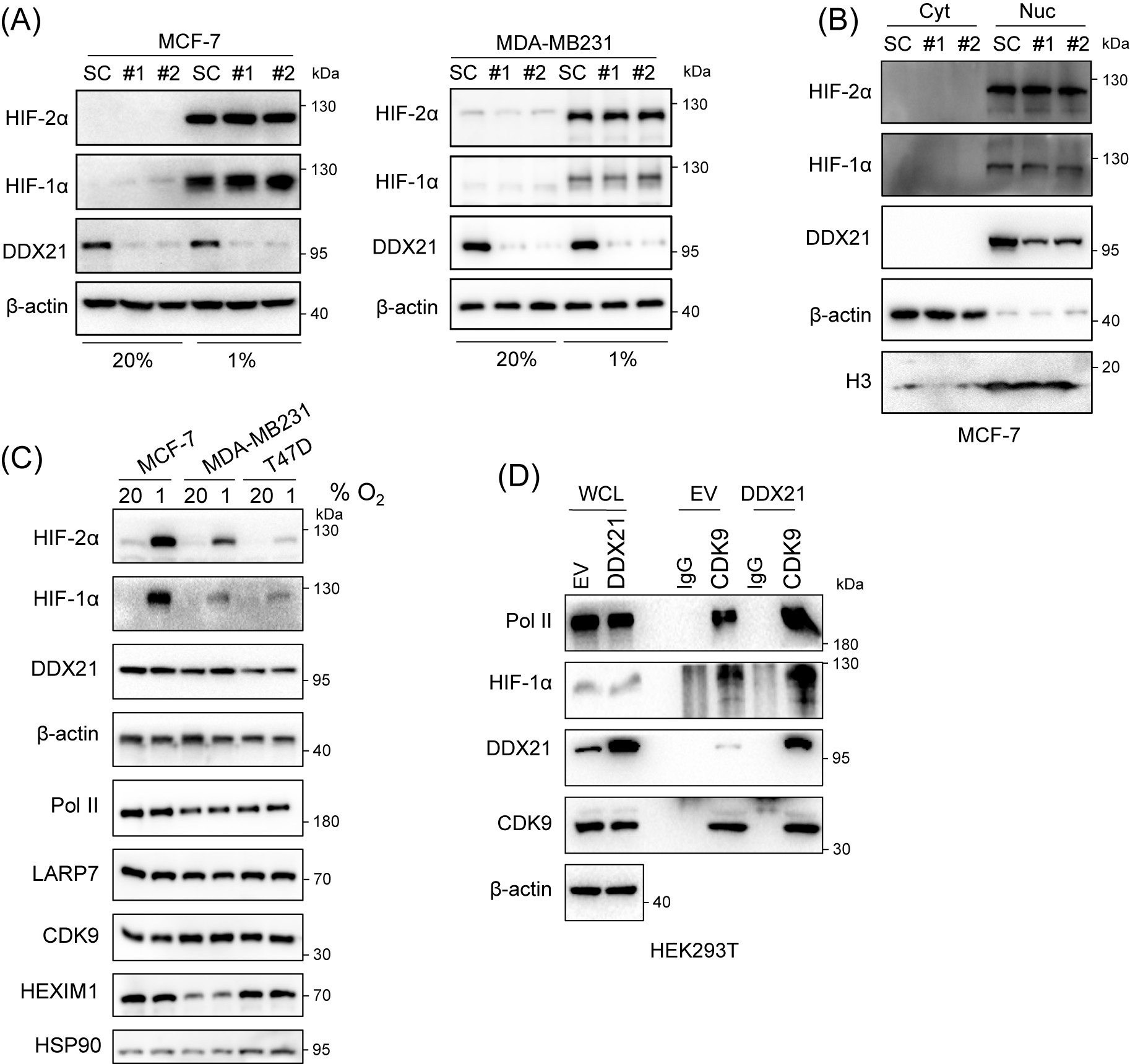


**Figure S4. DDX21 promotes CDK9 binding to HIF-1α.**

(A-B) Detection of protein levels of HIF-1α and HIF-2α in DDX21-KD MCF-7 cells and MDA-MB231 cells.

(B) Cytoplasmic and nuclear proteins were isolated from control cells and DDX21-knockdown cells for the detection of the subcellular localization of HIF-1α and HIF-2α.

(C) Assessment of DDX21, CDK9, HEXIM1, LARP7, RNA PII protein expression in different breast cancer cell lines following 24-hour hypoxic treatment.

(D) Co-IP experiments were performed in control and DDX21-overexpressing HEK-293T cells to investigate the interaction between CDK9 and HIF-1α and RNA Pol II.


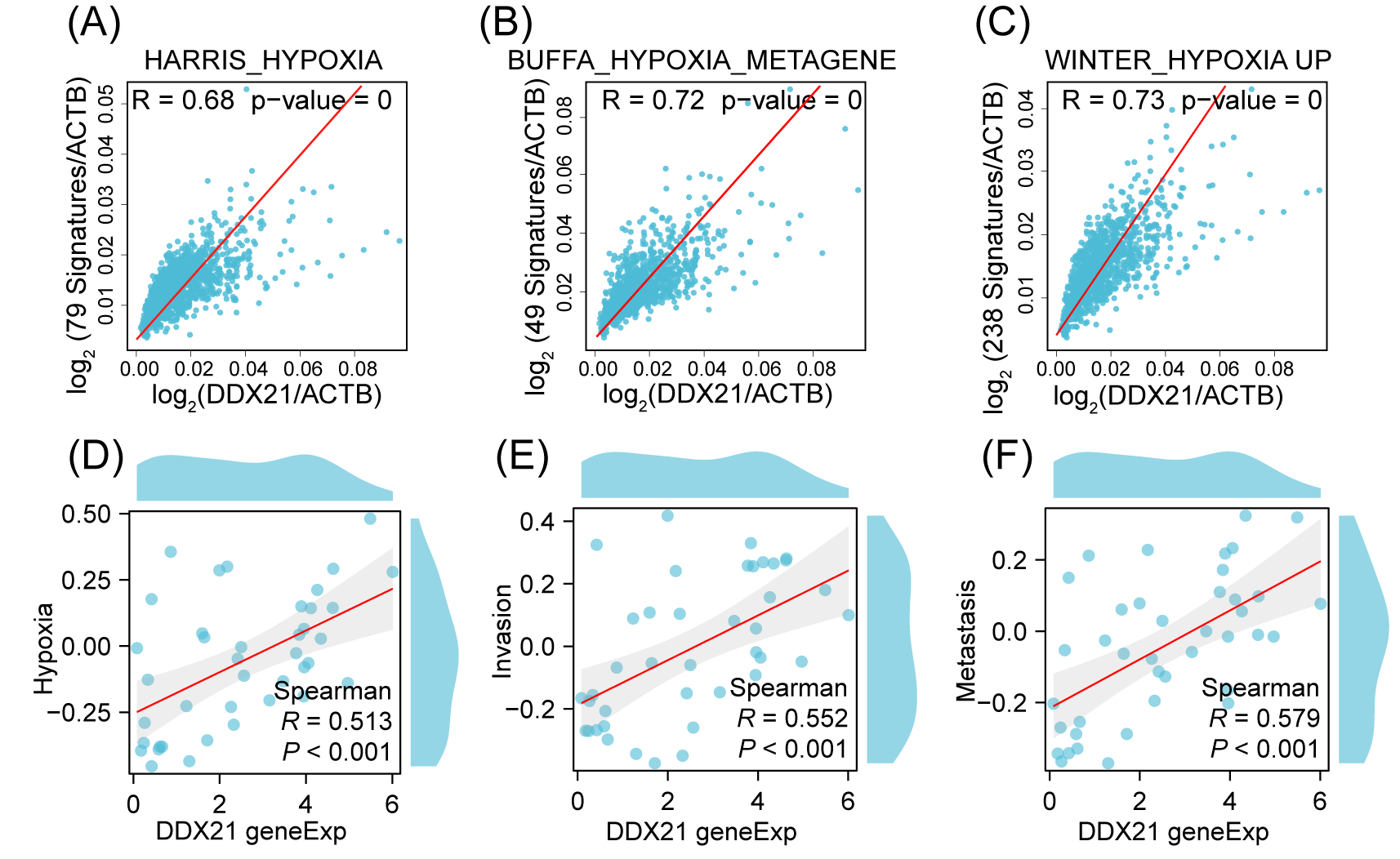


**Figure S5. Correlation between DDX21 expression and the hypoxia signature, biological processes.**

(A-C) Correlation analysis between DDX21 expression and the hypoxia signature based on HARRIS_HYPOXIA gene set (A), BUFFA_HYPOXIA_METAGENE gene set (B), and WINTER_HYPOXIA gene set (C).

(D-F) Analyze the correlations between DDX21 expression and biological processes related to Hypoxia, Invasion and Metastasis using the single-cell transcriptome data from CancerSEA.


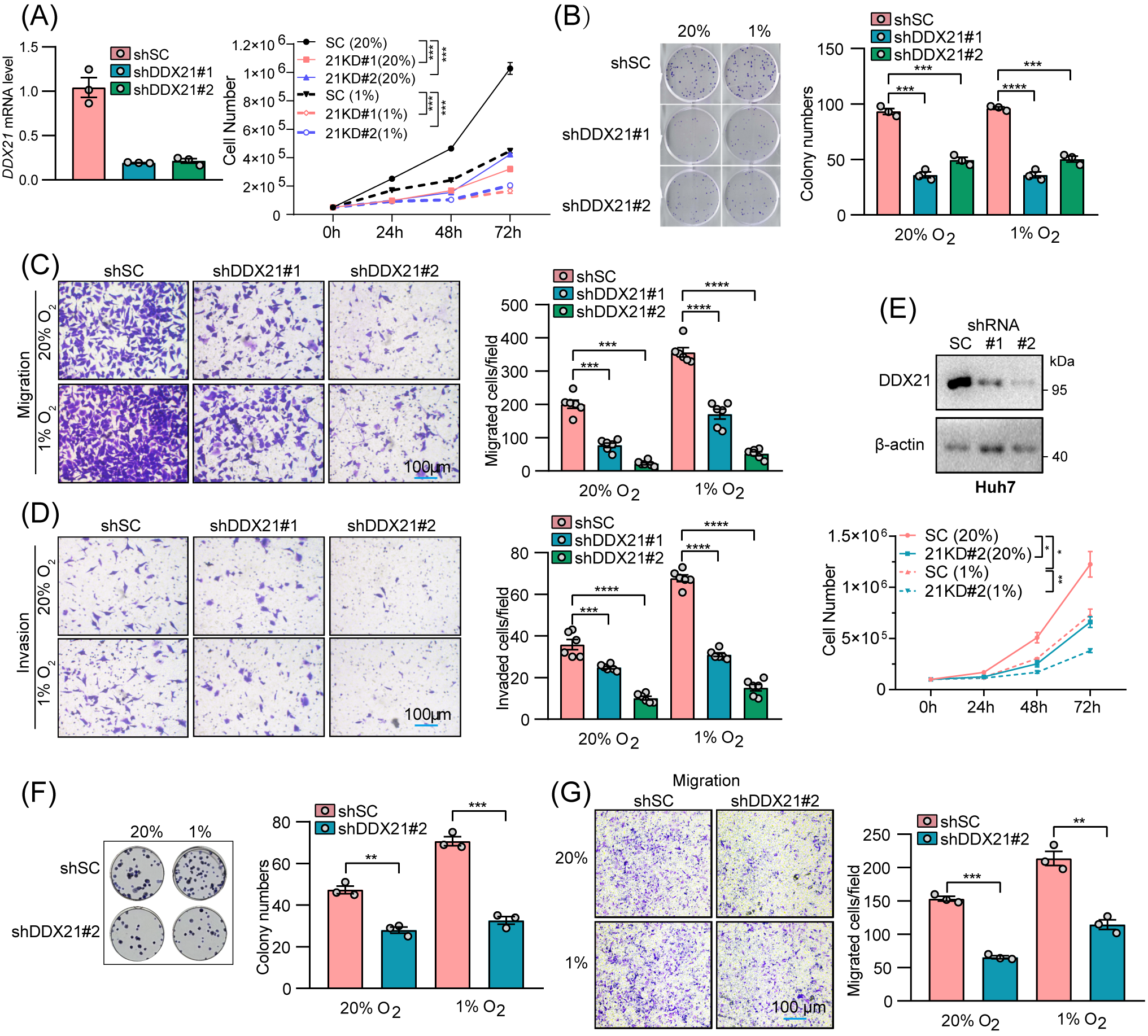


**Figure S6. DDX21 promotes the growth and migration of breast cancer and hepatocellular carcinoma cells in vitro.**

(A) Identification of MCF-7 DDX21 knockdown cell lines and evaluation of the effect of DDX21 knockdown on MCF-7 cell proliferation. (n=3, mean ± SEM, ***P<0.001).

(B) Clonogenic assays to evaluate the effect of DDX21 knockdown on cell survival. (n=3, mean ± SEM, ****P<0.0001)

(C-D) Evaluate the effects of DDX21 knockdown on tumor cell migration (C) and invasion (D). (n=5, mean ± SEM, ***P<0.001, ****P<0.0001)

(E) Identification of Huh7 DDX21 knockdown cell lines and evaluation of the effect of DDX21 knockdown on Huh7 cell proliferation. (n=3, mean ± SEM, *P<0.05, **P<0.01)

(F) Clonogenic assays to evaluate the effect of DDX21 knockdown on Huh7 cell survival. (n=3, mean ± SEM, **P<0.01, ***P<0.001)

(G) Evaluate the effects of DDX21 knockdown on Huh7 cell migration. (n=3, mean ± SEM, **P<0.01, ***P<0.001)


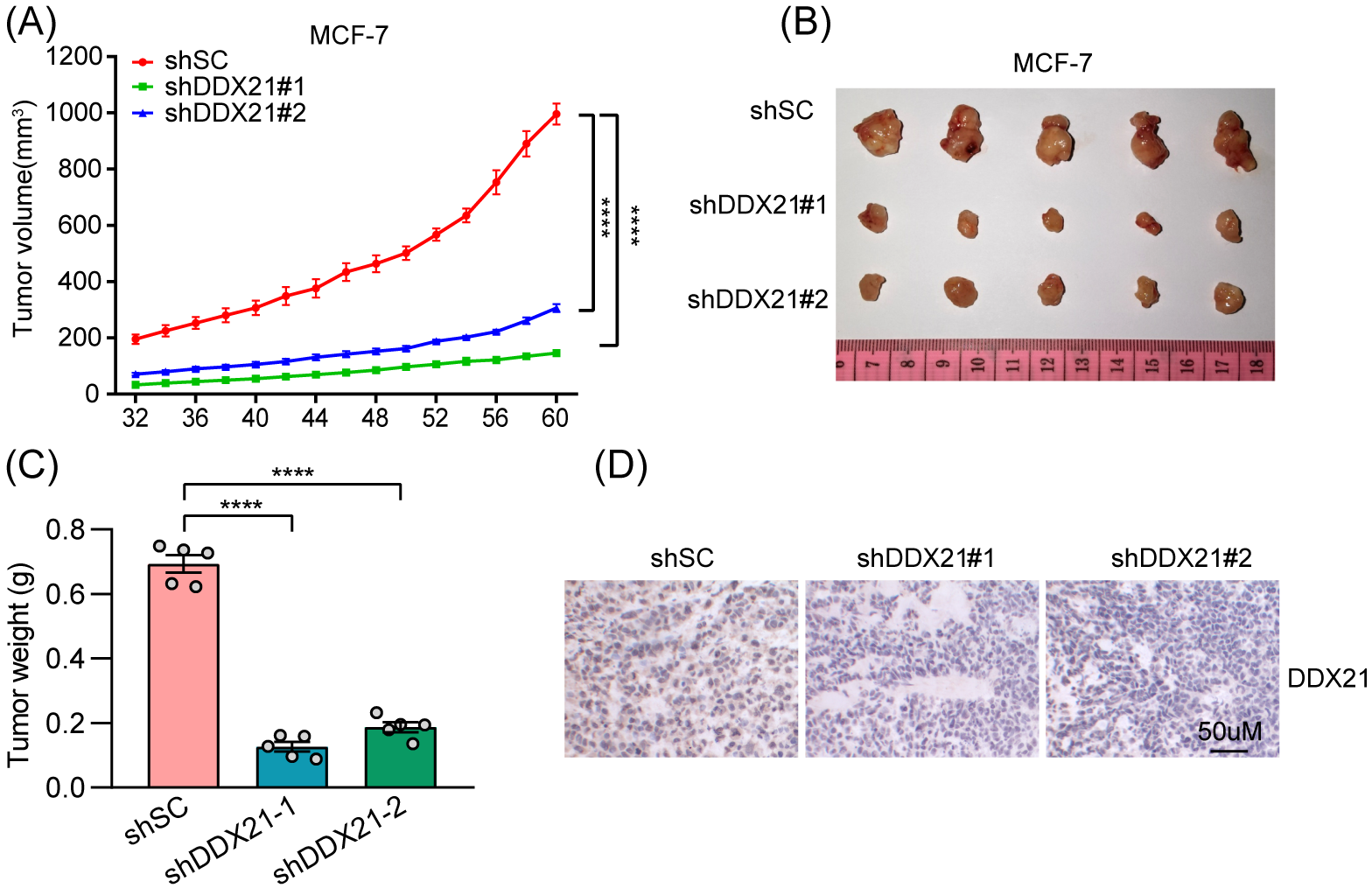


**Figure S7. DDX21 promotes breast cancer growth.**

(A-C) Growth curves(A), tumor size (B) and weight (C) of MCF-7 cells (shSC) and DDX21 knockdown MCF-7 cells (shDDX21#1 and shDDX21#2). (n=5, mean ± SEM, ****P<0.0001)

(D) IHC staining of tumor tissues with DDX21.


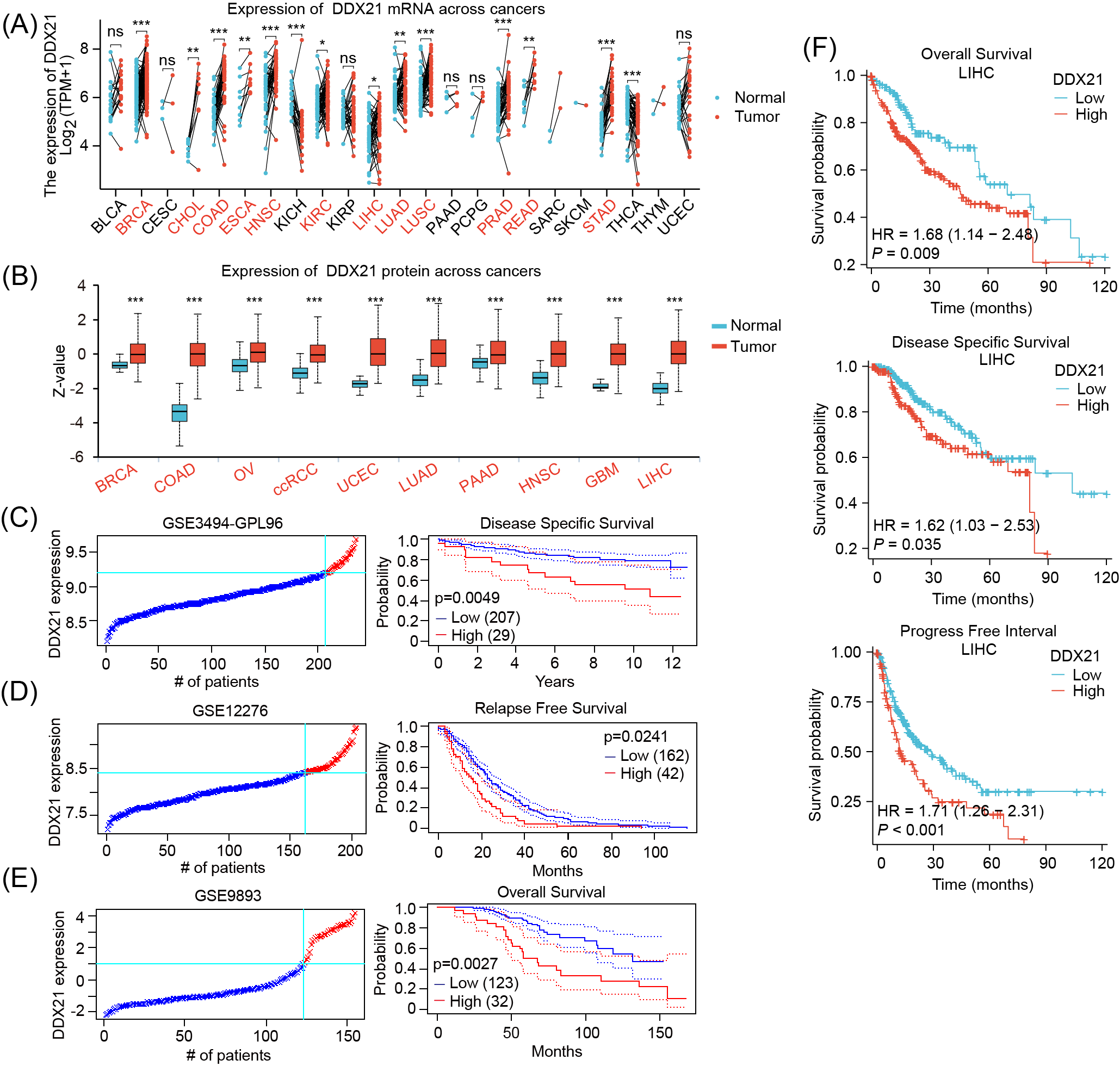


**Figure S8. Patients with high expression of DDX21 have poorer prognosis.**

(A) DDX21 mRNA expression levels across multiple cancer types using data from TCGA database.

(B) Expression of DDX21 protein across cancers based on the CPTAC dataset via the UALCAN platform.

(C-E) Correlation between DDX21 expression levels and breast cancer patient survival in GEO database.

(F) Associations between DDX21 expression and survival outcomes in LIHC.

**Tables S1** Primers for qPCR analysis

| Gene |  | Sequence |
| --- | --- | --- |
| IGFBP1 | F | 5’-TTGGGACGCCATCAGTACCTA-3’ |
|  | R | 5’-TTGGCTAAACTCTCTACGACTCT-3’ |
| TGFBI | F | 5’-CTTCGCCCCTAGCAACGAG-3’ |
|  | R | 5’-TGAGGGTCATGCCGTGTTTC-3’ |
| PCK1 | F | 5’-TTGAGAAAGCGTTCAATGCCA-3’ |
|  | R | 5’-CACGTAGGGTGAATCCGTCAG-3’ |
| APLN | F | 5’-GTCTCCTCCATAGATTGGTCTGC-3’ |
|  | R | 5’-GGAATCATCCAAACTACAGCCAG-3’ |
| WNT1 | F | 5’-CGATGGTGGGGTATTGTGAAC-3’ |
|  | R | 5’-CCGGATTTTGGCGTATCAGAC-3’ |
| WNT10B | F | 5'-GTGAGCGAGACCCCACTATG-3’ |
|  | R | 5’-CACTCTGTAACCTTGCACTCATC-3’ |
| SLC2A3 | F | 5’-TTCGTCTCTAGCCTGCACTG-3’ |
|  | R | 5’-ACACAACTTCTCCGGGTGAC-3’ |
| 18S | F | 5’-CGGCGACGACCCATTCGAAC-3’ |
|  | R | 5’-GAATCGAACCCTGATTCCCCGTC-3’ |
| DDX21 | F | 5’-GAGGAGCCATCTCAAAATGACA-3’ |
|  | R | 5’-GGGTTACAGTCCGGTTCAGG-3’ |
| ACTB | F | 5’-CATGTACGTTGCTATCCAGGC-3’ |
|  | R | 5’-CTCCTTAATGTCACGCACGAT-3’ |

**Tables S2** Primers for DDX21 FL and domain cloning

| DDX21 domain |  | Sequence |
| --- | --- | --- |
| 1-216AA | F | 5’-ATCACCGGTATGCCGGGAAAACTCCGTAGT-3’ |
|  | R | 5’-ATCGCGGCCGCTTATGTCTTTGCTTGTATAGG-3’ |
| 1-428AA | F | 5’-ATCACCGGTATGCCGGGAAAACTCCGTAGT-3’ |
|  | R | 5’-ATCGCGGCCGCTTACCCAATAACTGCTGCCCT-3’ |
| 429-573AA | F | 5’-ATCACCGGTGATGTCATCCGAGTATATAGTG-3’ |
|  | R | 5’-ATCGCGGCCGCTTATTTTATTATTTCTGTTGC-3’ |
| 1-216AA+429-573AA | F | 5’-CAAGCAAAGACAGATGTCATCCGA-3’ |
|  | R | 5’-TCGGATGACATCTGTCTTTGCTTG-3’ |
| F574-783AA | F | 5’-ATCACCGGTGCTTCCAGCAAAGATGCCATC-3’ |
|  | R | 5’-ATCGCGGCCGCTTATTGACCAAATGCTTTACTG-3’ |
| DDX21-FL | F | 5’-ATCACCGGTATGCCGGGAAAACTCCGTAGT-3’ |
|  | R | 5’-ATCGCGGCCGCTTATTGACCAAATGCTTTACTG-3’ |

**Tables S3** Primers for promoter cloning

| Promoter |  | Sequence |
| --- | --- | --- |
| WNT1 promoter(-2kb) | F | 5’-GCAAGCTTATGAGAGCTAGAACTCAA-3’ |
|  | R | 5’-ATGCTAGCGAGCCGAGAGCTGACGGGTT-3’ |
| IGFBP1 promoter(-2kb) | F | 5’-ATAAGCTTTGAGCCACTGAGCCTGGC-3’ |
|  | R | 5’-ATGCTAGCGTGGCCGATGCTCGCTGGACA-3’ |

**Tables S4** shRNA or sgRNA oligos

| shRNA or sgRNA | | Sequence |
| --- | --- | --- |
| HIF-1α sgRNA | 1α-F | 5’-CACCGCCATCAGCTATTTGCGTGTG-3’ |
|  | 1α-R | 5’-AAACCACACGCAAATAGCTGATGGC-3’ |
| HIF-2α sgRNA | 2α-F | 5’-CACCGGCTGATTGCCAGTCGCATGA-3’ |
|  | 2α-R | 5’-AAACTCATGCGACTGGCAATCAGCC-3’ |
| shDDX21-1 | 21-1F | 5’-CCGGGGTGGCTCCATAGCTTTATTTCTCGAGAAATAAAGC  TATGGAGCCACCTTTTT-3’ |
|  | 21-1R | 5’-AATTAAAAAGGTGGCTCCATAGCTTTATTTCTCGAGAAAT  AAAGCTATGGAGCCACC-3’ |
| shDDX21-2 | 21-2F | 5’-CCGGCCTGAGGTTGATTTGGTTATACTCGAGTATAACCAA  ATCAACCTCAGGTTTTT-3’ |
|  | 21-2R | 5’-AATTAAAAACCTGAGGTTGATTTGGTTATACTCGAGTAT  AACCAAATCAACCTCAGG-3’ |

**Tables S5** Ch-IP qPCR Primers

| Primers | Sequence |
| --- | --- |
| WNT1-1F | TACCACAGTGTGACACGC |
| WNT1-1R | GAGGGATGCAGAGTGTCT |
| WNT1-2F | CCTTCGTCAGAACAAGGAG |
| WNT1-2R | AGCGCACAAGTGGCTGTGT |
| IGFBP1-1F | CAGAGTTGCTCTTTCTTG |
| IGFBP1-1R | CTATCTGGAAGCCATACTG |
| IGFBP1-2F | CACGTCTGTCTCATCATC |
| IGFBP1-2R | ATCCCTGCTGCTCCCTTCA |
| RPL13A-F | GAGGCGAGGGTGATAGAG |
| RPL13A-R | ACACACAAGGGTCCAATTC |

**Tables S6** Hypoxia signatures according MsigDB

| Gene Set | genes | Ref |
| --- | --- | --- |
| HARRIS_HYPOXIA | ADM,AK3,ALDOA,ANGPT2,APEX1,BHLHE40,BIK,BNIP3,BNIP3L,CA12,CA9,CCL2,CCNG2,CD99,CDKN1A,CDKN1B,COL5A1,CP,CXCL8,DDIT3,EDN1,EDN2,ENO1,ENPEP,EPAS1,EPO,F3,FGF3,FLT1,FOS,FTL,GAPDH,HDAC9,HGF,HIF1A,HK1,HK2,HMOX1,IGF2,IGFBP1,IGFBP2,IGFBP3,IL6,JUN,L1CAM,LDHA,LRP8,MIF,MMP13,NFKB1,P4HA1,PDGFB,PFKL,PFKP,PGF,PGK1,PKM,PLAUR,PRPS1,PTGS2,RP1,SAT1,SLC2A1,SLC2A3,SPP1,STC1,TAGLN,TEK,TF,TFF3,TFRC,TGFA,TGFB1,TGFB3,TGM2,TH,TXN,VEGFA,VIM,XRCC5,XRCC6 | [Pubmed 11902584](https://pubmed.ncbi.nlm.nih.gov/11902584) |
| BUFFA_HYPOXIA_METAGENE | ACOT7,ADM,AK4,ANKRD37,ANLN,BNIP3,CA9,CDKN3,CHCHD2,CORO1C,CTSV,DDIT4,ENO1,ESRP1,GAPDH,GPI,HILPDA,HK2,KIF20A,KIF4A,LDHA,LRRC42,MAD2L2,MAP7D1,MCTS1,MIF,MRGBP,MRPL13,MRPL15,MRPS17,NDRG1,P4HA1,PFKP,PGAM1,PGK1,PNP,PSMA7,PSRC1,SEC61G,SHCBP1,SLC16A1,SLC25A32,SLC2A1,TPI1,TUBA1A,TUBA1C,TUBB6,UTP11,VEGFA,YKT6 | [Pubmed 20087356](https://pubmed.ncbi.nlm.nih.gov/20087356) |
| WINTER_HYPOXIA_UP | ABCB1,ACAT1,ADM,ADORA2B,AK2,AK3,ALDH1A1,ALDH1A3,ALDOA,ALDOC,ANGPT2,ANGPTL4,ANXA1,ANXA2,ANXA5,ARHGAP5,ARSL,ART1,BACE2,BATF3,BCL2L1,BCL2L2,BHLHE40,BHLHE41,BIK,BIRC2,BNIP3,BNIP3L,BPI,BTG1,CA12,CA9,CALD1,CCNG2,CCT6A,CD99,CDK1,CDKN1A,CDKN1B,CITED2,CLK1,CNOT7,COL4A5,COL5A1,COL5A2,COL5A3,CP,CTSD,CXCL8,CXCR4,DDIT3,DDIT4,DELEC1,DKC1,DR1,EDN1,EDN2,EFNA1,EGF,EGR1,EIF4A3,ELF3,ELL2,ENG,ENO1,ENO3,ENPEP,EPO,ERRFI1,ETS1,F3,FABP5,FGF3,FKBP4,FLT1,FN1,FOS,FTL,GAPDH,GBE1,GLRX,GPI,HAP1,HBP1,HDAC1,HDAC9,HERC3,HERPUD1,HGF,HIF1A,HILPDA,HK1,HK2,HLA-DQB1,HMOX1,HMOX2,HSP90B1,HSPA5,HSPD1,HSPH1,HYOU1,ICAM1,ID2,IFI27,IGF2,IGFBP1,IGFBP2,IGFBP3,IGFBP5,IL6,INSIG1,IRF6,ITGA5,JUN,KDR,KRT14,KRT18,KRT19,LDHA,LDHB,LEP,LGALS1,LONP1,LOX,LRP1,MAP4,MET,MIF,MMP13,MMP2,MMP7,MPI,MT-CO1,MT-CO2,MT1L,MTL3P,MUC1,MXI1,NDRG1,NFIL3,NFKB1, NFKB2,NOS1,NOS2,NOS2P1,NOS2P2,NOS3,NR3C1,NR4A1,NSG1,NT5E,ODC1,P4HA1,P4HA2,PAICS,PDGFB,PDK3,PFKFB1,PFKFB3,PFKFB4,PFKL,PGAM1,PGF,PGK1,PGK2,PGM1,PIM1,PIM2,PKM,PLAU,PLAUR,PLIN2,PLOD2,PNN,PNP,POLM,PPARA,PPAT,PROK1,PSMA3,PSMD9,PTGS1,PTGS2,QSOX1,RBPJ,RELA,RIOK3,RNASEL,RPL36A,RRP9,SAT1,SERPINB2,SERPINE1,SGSM2,SIAH2,SIN3A,SIRPA,SLC16A1,SLC16A2,SLC20A1,SLC2A1,SLC2A3,SLC3A2,SLC6A10P,SLC6A16,SLC6A6,SLC6A8,SORL1,SPP1,SRSF6,STC2,SYT7,TBPL1,TCEAL1,TEK,TF,TFF3,TFRC,TGFA,TGFB1,TGFB3,TGFBI,TGM2,TH,THBS1,THBS2,TIMM17A,TNFAIP3,TP53,TPBG,TPD52,TPI1,TXN,TXNIP,UMPS,VEGFA,VEGFB,VEGFC,VIM,VPS11,VPS51,XRCC6,ZNRD2 | [Pubmed 17409455](https://pubmed.ncbi.nlm.nih.gov/17409455) |

**Tab S7** Key resources table

| REAGENT or RESOURCE | SOURCE | IDENTIFIER |
| --- | --- | --- |
| **Anti-bodies** | | |
| Anti-DDX21 | Novus | Cat# NB100-1718 |
| Anti-DDX21 | Proteintech | Cat# 66925-1-Ig |
| Anti-HIF1α | Bethyl | Cat# A303-907A |
| Anti-HIF2α | Bethyl | Cat# A700-003 |
| Anti-HEXIM1 | Bethyl | Cat# A303-113A |
| anti-LARP7 | Bethyl | Cat# A303-723A |
| Anti-CDK9 | [Santa Cruz](https://www.scahealth.com/home?lang=en&sessionShare=true) | Cat# sc-13130 |
| Anti-Cleaved-Caspase-3， | Servicebio | Cat# GB11532 |
| Anti-RNA polymerase II CTD | Abcam | Cat# ab26721 |
| Anti-RNA polymerase II CTD (phospho S2) | Abcam | Cat# ab5095 |
| Anti-β-actin (Clone 2D4H5) | Proteintech | Cat# 66009-1 |
| HA Tag Recombinant antibody | Proteintech | Cat# 81290-1-RR |
| FLAG (DYKDDDDK) Tag Mouse mAb | Vazyme | Cat# RA1003-01 |
| Normal Mouse IgG | Cell Signaling Technology | Cat# 68860 |
| Normal Rabbit IgG | Cell Signaling Technology | Cat# 2729S |
| anti-Myc | Proteintech | Cat# 60003-2-Ig |
| anti-SLC2A3 | Proteintech | Cat# 20403-1-AP |
| Anti-His | Proteintech | Cat# 66005-1-Ig |
| GST Tag Monoclonal Antibody | Invitrogen | Cat# MA4-004 |
| **Chemicals, peptides, and recombinant proteins** | | |
| Lipofectamine 3000 | Invitrogen | Cat# L3000-015 |
| TRIzol Reagent | ThermoFisher | Cat# 15596018 |
| EDTA-free protease inhibitor cocktail | Roche | Cat# 11697498001 |
| SureBeads™ Protein A Magnetic Beads | Bio-Rad | Cat# 1614013 |
| Anti-FLAG^®^ M2 magnetic beads | Sigma | Cat# M8823 |
| Puromycin | Sigma | Cat# P7255 |
| Ampicillin | Sangon Biotech | Cat# A100339 |
| Magnetic Stand | MedChemExpress | Cat# HY-K0200 |
| Hypoxia Chamber | STEMCELL TECHNOLOGIES | Cat# 27310 |
| BsmBI-v2 | NEB | Cat# R0739S |
| Stbl3 Competent Cells | Genesand | Cat# XK002-01 |
| T4 DNA Ligase | NEB | Cat# M0202S |
| AgeI-HF^®^ Restriction Enzymes | NEB | Cat# R3552S |
| EcoRI- HF^®^ Restriction Enzymes | NEB | Cat# R3101S |
| FBS | Gibco | Cat# A5256701 |
| DMEM | Gibco | Cat# C11995500BT |
| RPMI-1640 | Gibco | Cat# C11875500BT |
| Trypsin | Gibco | Cat# 25200072 |
| Penicillin-Streptomycin | Gibco | Cat# 15140122 |
| Matrigel | Corning | Cat# 356234 |
| BCA Protein Assay Kit | Beyotime | Cat# P0006 |
| Dual-Luciferase® Reporter Assay System | Promega | Cat# E1910 |
| Pierce™ Magnetic ChIP Kit | Pierce | Cat# 26157 |
| Nuclear protein and cytoplasmic protein extraction kit | Beyotime | Cat# P0027 |
| Glutathione Sepharose™ 4B | Cytiva | Cat# 17‑0756‑01 |
| HisSep Ni-NTA MagBeads | YEASON | Cat# 20561ES08 |
| IPTG | MCE | Cat# HY-15921 |
| BL21（DE3）Chemically Competent Cell | Tsingke | Cat# TSC-E01 |
| Hyperactive pG-MNase CUT&RUN Assay Kit for Illumina | Vazyme | Cat# HD102 |

**Experimental models: Cell lines**

| MCF-7 | ATCC | CRL-3435 |
| --- | --- | --- |
| MDA-MB231 | ATCC | HTB-26 |
| HEK293T | ATCC | CRL-11268 |
| HeLa | ATCC | CRM-CCL-2 |
| T47D | ATCC | HTB-133 |
| SUM-159 | ATCC | CVCL_5423 |
| Huh7 | CCLV | CCLV-RIE 1079 |
